# Hypernova: a faster and brighter light-producing deoxyribozyme

**DOI:** 10.1093/nar/gkag1143

**Published:** 2026-11-13

**Authors:** Katerina Svehlova, Jaroslav Kurfürst, Martin Jakubec, Edward A Curtis

**Affiliations:** Institute of Organic Chemistry and Biochemistry of the Czech Academy of Sciences, Prague 166 10, Czech Republic; Institute of Organic Chemistry and Biochemistry of the Czech Academy of Sciences, Prague 166 10, Czech Republic; Department of Informatics and Chemistry, University of Chemistry and Technology, Prague 166 28, Czech Republic; Institute of Organic Chemistry and Biochemistry of the Czech Academy of Sciences, Prague 166 10, Czech Republic; Department of Genetics and Microbiology, Faculty of Science, Charles University in Prague, Prague 128 44, Czech Republic; Institute of Organic Chemistry and Biochemistry of the Czech Academy of Sciences, Prague 166 10, Czech Republic

## Abstract

As a functional nucleic acid motif is optimized, better variants become less abundant in sequence space and significantly more difficult to identify. Here, we used two types of libraries to discover an improved variant of a previously optimized light-producing deoxyribozyme called Supernova. One library was designed in such a way that most mutations were consistent with the sequence requirements and secondary structure of this catalytic motif. The second contained short stretches of random sequence in place of AAAA spacers previously introduced in minimization experiments. Catalytically active sequences were isolated from each library using single and multiple round selections. Mutations from the best deoxyribozymes were then combined to yield an improved variant containing 12 mutations, which we named Hypernova. The rate of Hypernova at subsaturating substrate concentrations was up to 97-fold higher than that of Supernova, and its initial rate of light production was up to 30-fold faster. In addition, sensors constructed using Hypernova outperformed those made from Supernova in three different assays. These results indicate that Hypernova can enhance the performance of light-producing deoxyribozyme sensors for applications such as diagnostics and high-throughput screening.

## Graphical Abstract

**Graphical Abstract:**
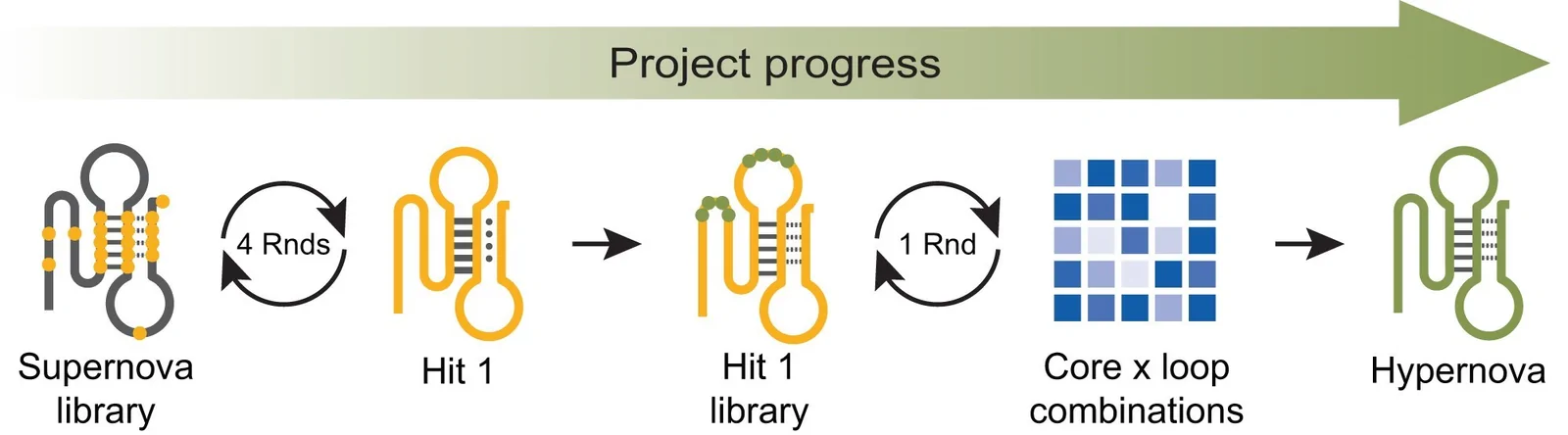

## Introduction

Functional nucleic acids such as aptamers, ribozymes, and deoxyribozymes are useful tools for a wide variety of applications [1–4]. Examples include aptamers that can function as substitutes for protein antibodies [2], RNA-cleaving ribozymes and deoxyribozymes for manipulation of gene expression [5, 6] and detection of transcripts with modified nucleotides [7, 8], polymerase ribozymes that can synthesize smaller ribozymes [9], fluorescent aptamers as genetic reporters [10–12], allosteric ribozymes and deoxyribozymes as sensors [13–16], and deoxyribozymes that generate chemiluminescent [17, 18], fluorescent [19–21], and chromogenic signals [22–24] as diagnostic tools. Such motifs are usually identified using methods of artificial evolution such as SELEX and *in vitro* selection. In a typical selection experiment, a library of random DNA or RNA sequences is iteratively enriched for molecules with a phenotype of interest until they are abundant enough to be detected by sequencing. Newer approaches have started to explore the use of structured rather than random sequence or randomly mutagenized libraries [20, 25, 26] and single-step rather than multiple-round selections [27, 28] to increase efficiency and throughput. Although both DNA and RNA motifs are useful tools, DNA offers unique advantages for certain applications. These include high chemical stability and the possibility of rapid and inexpensive synthesis using solid-phase methods [2–4].

Although selection is a powerful and general method, functional sequences isolated from random sequence libraries are often not active enough to be useful for practical applications. While improved variants can typically be isolated by random mutagenesis of a functional sequence followed by reselection [29, 30], further optimization can be challenging. One reason is that as a sequence is optimized, the frequency of improved variants in sequence space progressively decreases, making further improvement more and more difficult [31, 32]. This limitation is compounded by sampling problems. Libraries in selection experiments are generally limited to ~10^16^ sequences, which corresponds to the number of possible variants of a ~26-nucleotide long sequence. Most functional motifs are longer than this, however, and therefore cannot be comprehensively mapped using standard methods of random mutagenesis and selection. These considerations highlight the difficulties of improving functional DNA and RNA motifs, especially those that have been previously optimized.

In this study, we set out to identify improved variants of a catalytic DNA motif called Supernova. This deoxyribozyme transfers the phosphate group from the 1,2-dioxetane substrate CDP-Star to its own 5′-hydroxyl group, which triggers production of light by a chemically initiated electron exchange luminescence (CIEEL) mechanism [33]. We previously optimized the sequence of this motif by random mutagenesis and reselection [17], which yielded a variant with a catalytic efficiency six-fold higher than that of the initial isolate (we refer to this improved variant as Supernova, and use the term Supernova motif to refer to the set of all sequences with this structure and function). Here, we isolated significantly better variants of Supernova using two new types of libraries. One was synthesized using a new approach and made up of sequences consistent with the secondary structure and sequence requirements of this motif [20, 25]. Base triples were encoded in such a way that they were rarely disrupted, and each of the three canonical possibilities (CGG, TAA, and TAT) could occur at each position in the triple helix of Supernova. A second library contained short patches of random sequence in place of AAAA spacers that were introduced in previous minimization experiments. The sequence of each AAAA spacer was arbitrary, and we hypothesized that systematic optimization might yield better variants. Deoxyribozymes were isolated using both single-step and multiple-round selections and analyzed using several different methods to find a metric that maximizes the correlation between frequency and catalytic activity. Mutations from the most active variants from each library were then combined to yield even better variants. The most active deoxyribozyme identified, which we named Hypernova, catalyzed self-phosphorylation with a rate up to 97-fold faster than that of Supernova, and generated light 30-fold faster under some conditions. It could also be used to detect target molecules, and significantly outperformed Supernova in three different assays. We anticipate that Hypernova will be useful for the next generation of light-producing deoxyribozyme sensors.

## Materials and methods

### Oligonucleotides

HPLC-purified oligonucleotides were ordered from Eurofins Genomics GmbH. The sequences of all oligonucleotides used in this study are listed in Supplementary Table S1.

### Library synthesis and preparation

Libraries were ordered from Integrated DNA Technologies and purified by 6% urea–PAGE. In the case of the secondary structure library, 16 degenerate oligonucleotides were mixed in equal amounts to obtain the final library. Both the secondary structure library and the spacer library were characterized by high-throughput sequencing. This showed that expected and observed nucleotide frequencies were similar at variable positions (Supplementary Figs S1 and S2).

### *In vitro* selection of the secondary structure library

The secondary structure library was mixed with the blocking oligonucleotide (Rev1) in water, heated at 65°C for 2 min, and cooled at room temperature for 5 min. The mixture was then incubated for 100 s with CDP-Star at the following final concentrations: 1 μM DNA library, 1.5 μM Rev1, 1× selection buffer (50 mM HEPES pH 7.4, 20 mM KCl, 650 μM ZnCl_2_), and 125 μM CDP-Star. The reaction was stopped by adding EDTA to a final concentration of 20 mM. After this, the DNA was ethanol precipitated and ligated to a short oligonucleotide (Fwd1) using a splint (Splint1) and T4 DNA ligase (Jena Bioscience). The ligation reaction was incubated for 5 min with all oligonucleotides at a concentration of 2.5 μM. Molecules were then separated by 6% urea–PAGE, and those of a length of 88 nucleotides (nt) were purified. This purification step was repeated. The isolated molecules were then amplified by PCR using Q5 Hot Start High-Fidelity Polymerase (NEB) and the Fwd1r and Rev1p primers. The Fwd1r primer contained a single RNA linkage at its 3′ end and the Rev1p primer contained a phosphate at its 5′ end. After cleaning up the reaction using the NucleoSpin Gel and PCR Clean-up kit (Macharey-Nagel), the reverse strand was digested using λ-exonuclease (NEB). After another purification with the NucleoSpin Gel and PCR Clean-up kit, the remaining sense strands were base hydrolyzed and purified by 6% urea–PAGE to regenerate the single-stranded DNA library. During each round, the isolated molecules were amplified by PCR using Q5 Hot Start High-Fidelity Polymerase (NEB) and the Fwd1 and Rev1 primers, and sent for sequencing at Eurofins Genomics GmbH.

### *In vitro* selection of the randomly mutagenized control library

The randomly mutagenized control library was mixed with the blocking oligonucleotide (Rev1) in water, heated at 65°C for 2 min, and cooled at room temperature for 5 min. The mixture was then incubated for 100 s with CDP-Star at the following final concentrations: 1 μM DNA library, 1.5 μM Rev1, 1× selection buffer, and 125 μM CDP-Star. The reaction was stopped by adding EDTA to a final concentration of 20 mM. After this, the DNA was ethanol precipitated and ligated to a short oligonucleotide (Fwd1) using a splint (Splint1) and T4 DNA ligase (Jena Bioscience). The ligation reaction was incubated for 5 min, and all oligonucleotides were at a concentration of 2.5 μM. Molecules were then separated by 6% urea–PAGE, and those of the length of 88 nt were purified. This purification step was repeated. The isolated molecules were amplified by PCR using Q5 Hot Start High-Fidelity Polymerase (NEB) and the Fwd1 and Rev1 primers, and sent for sequencing at Eurofins Genomics GmbH.

### *In vitro* selection of the spacer library

The spacer library was mixed with the blocking oligonucleotide (Rev2) in water and heated at 65°C for 2 min and cooled at room temperature for 5 min. The mixture was then incubated for 10 s with CDP-Star at the following final concentrations: 1 μM DNA library, 1.5 μM Rev2, 1× selection buffer, and 125 μM CDP-Star. The reaction was stopped by adding EDTA to a final concentration of 20 mM. After this, the DNA was ethanol precipitated and ligated to a short oligonucleotide (Fwd2) using a splint (Splint2) and T4 DNA ligase (Jena Bioscience). The ligation reaction was incubated for 5 min, and all oligonucleotides were at a concentration of 2.5 μM. Molecules were then separated by 6% urea–PAGE, and those of the length of 88 nt were purified. This purification step was repeated. The isolated molecules were amplified by PCR using Q5 Hot Start High-Fidelity Polymerase (NEB) and the Fwd2 and Rev2 primers, and sent for sequencing at Eurofins Genomics GmbH.

### Activity analysis by ligation

HPLC-purified oligonucleotides corresponding to individual deoxyribozymes from evolved libraries were ordered from Eurofins Genomics GmbH. Each deoxyribozyme was mixed with water, heated at 65°C for 2 min and cooled at room temperature for 5 min. Afterwards, 5× selection buffer and CDP-Star were added. Final concentrations were 1 μM deoxyribozyme, 1× selection buffer, and CDP-Star at the indicated concentration. The reaction was incubated for a specified time at room temperature in the dark and stopped by the addition of EDTA to a final concentration of 20 mM. The reaction was then cleaned up using SigmaSpin Sequencing Reaction Clean-Up columns (Sigma–Aldrich) and ethanol precipitated. Reacted deoxyribozyme was then ligated to a short oligonucleotide as in the selection except that a ligation time of 30 min was used. Reacted and unreacted molecules were then separated by 6% urea–PAGE, and the ligation yield was analyzed using a gel imager (Amersham Typhoon) and a densitometry tool ImageQuant TL (GE Healthcare LifeSciences).

### Analysis of light production

HPLC-purified oligonucleotides corresponding to individual deoxyribozymes from evolved libraries were ordered from Eurofins Genomics. Light production was assessed as follows: the deoxyribozyme being tested was mixed with water, heated at 65°C for 2 min, and cooled at room temperature for 5 min. Afterwards, 5× selection buffer was added, and samples were transferred to a white half-area 96-well plate (Corning). CDP-Star was added, and chemiluminescence was immediately measured for 1 h using a Tecan Spark plate reader (Tecan Group). Final concentrations were 1 μM deoxyribozyme, 1× selection buffer, and CDP-Star at the indicated concentration.

### Next generation sequencing and data analysis

Secondary structure libraries were sequenced by Eurofins Genomics using amplicon paired-end sequencing runs. Loop libraries were sequenced by Novogene using amplicon paired-end sequencing runs. Data processing and analysis was done using custom bash and python scripts. Raw data, processed data, processing scripts and analysis notebooks are available at https://doi.org/10.6084/m9.figshare.34097340.

### Self-phosphorylation kinetics and analysis

Reaction kinetics were characterized using a ligation assay. To do this, the deoxyribozyme being tested was mixed with water, incubated at 65°C for 2 min, and cooled at room temperature for 5 min. Afterwards, 5× selection buffer and CDP-Star were added. Final concentrations were 1 μM deoxyribozyme, 1× selection buffer, and CDP-Star at the indicated concentration. Some reactions were performed using a variant of Hypernova containing a 3′ AAA linker followed by a Cy3 label (Eurofins Genomics GmbH). In these cases, the final deoxyribozyme concentration was 40 nM. Reactions were stopped by adding EDTA to a final concentration of 25 mM at selected time points of the reaction. Samples were then cleaned up, ethanol precipitated, and ligated as described in the section Activity analysis by ligation. The signal at time zero was subtracted from each sample. Rates were determined using Prism 11 (Graphpad Software), as a one-phase association of a nonlinear regression (curve fit), using the equation P = F_max_ × (1 – e^−^*^kt^*), where P is the percent ligated, F_max_ is the maximum percent ligated, *k* is the rate, and *t* is time. The *k*_cat_/K_M_ value of Cy3-labeled Hypernova was determined using Prism 11 as a simple linear regression fit of rates (each determined at least three times as described above) at 1 μM, 3.3 μM, 10 μM, and 33 μM CDP-Star using the equation *k*_obs_ = (*k*_cat_/K_M_) × [S] (which applies when K_M_ >> S). Quality of fits were assessed by R^2^. In all cases, error values reported in this manuscript indicate one standard deviation. The error in the *k*_cat_/K_M_ value of Hypernova was determined using GraphPad, while other error values were calculated using the STDEVA function of Excel or manually using standard propagation of error formulas.

### RNase sensor experiments

HPLC-purified oligonucleotides were mixed with water, heated at 65°C for 2 min, and cooled at room temperature for 5 min. Afterwards, 5× selection buffer and RNase A (Thermo Scientific) were added and the mixture was incubated at room temperature for 1 h. Next, samples were transferred to a white half-area 96-well plate (Corning), CDP-Star was added, and chemiluminescence was immediately measured for 1 h using a Tecan Spark plate reader. Final concentrations were 1 μM deoxyribozyme, 1× selection buffer, 500 nM RNase A, and 62.5 μM CDP-Star in a volume of 100 μl.

### DNase sensor experiments

HPLC-purified oligonucleotides were incubated with 1× DNase I Reaction Buffer and 2 U of DNase I (NEB) in a total volume of 20 μl at 37°C for 30 min. Next, water and 5× selection buffer were added and samples were transferred to a white half-area 96-well plate (Corning). CDP-Star was added, and chemiluminescence was immediately measured for 1 h using a Tecan Spark plate reader. Final concentrations were 1 μM deoxyribozyme, 1× selection buffer, 2 U of DNase I, and 62.5 μM CDP-Star in a volume of 100 μl.

### APOBEC3A sensor experiments

HPLC-purified oligonucleotides were mixed with water, heated at 65°C for 2 min, and cooled at room temperature for 5 min. The DNA-editing reaction was initiated by adding recombinant APOBEC3A in 2× A3A buffer. Final concentrations were 50 nM APOBEC3A, 1× A3A buffer (20 mM NaCl, 0.5% Triton, 7.5 mM Tris–HCl, pH 7.4), and 3.3 μM DNA in a total volume of 30 μl. Reactions were incubated at room temperature in the dark with moderate shaking for 20 min, after which APOBEC3A was inactivated by vortexing. Water and 5× selection buffer were then added to final concentrations of 1× selection buffer and 1 μM DNA. The samples were transferred to a white half-area 96-well plate, and the light-producing reaction was initiated by adding CDP-Star to a final concentration of 62.5 μM in a final volume of 100 μl.

### Calculation of signal-to-background ratios and initial rates of light production

Measurements from the light production assay were analyzed as described in Supplementary Fig. S3a–c. To determine signal-to-background ratios, a buffer value (measured in 1× selection buffer) was subtracted from the value of each sample at the respective time point. For all samples except the negative control (NC), the cumulative light production was calculated by adding up values (from which the buffer value had been subtracted) from all previous time points. Next, the average background signal per measurement (CDP-Star in 1× selection buffer) was calculated over the 60-min period. This value was used to calculate the background cumulative light production over the complete time course. Next, we divided the cumulative light value of each sample at each time point by the cumulative value of background. The final signal-over-background ratio is the maximum value over the 60-min experiment.

To determine the initial rates of light production, the data were processed as described above. Instead of dividing by NC, the first 2 minutes of cumulative light production of each sample and NC were fitted by linear regression and the slope of the reaction progress was determined (Supplementary Fig. S3a–c). These two methods yielded similar results (Supplementary Fig. S4).

## Results

### Design and synthesis of a secondary structure library based on Supernova

Supernova is a small catalytic DNA motif recently discovered in our group (Fig. 1a) [17, 18]. It transfers the phosphate group from the 1,2-dioxetane substrate CDP-Star to its own 5′ hydroxyl group, which triggers formation of a chemiluminescent intermediate and the production of light [33]. The initial isolate of the Supernova motif was originally isolated from a random sequence library and further optimized by random mutagenesis and reselection. This latter library likely contained all possible variants within ~4 mutations of the initial isolate, but provided little information about more distant variants (such as those in which each of the four base triples in Supernova has changed from CGG to another canonical triple such as TAA or TAT). To explore the sequence requirements of the Supernova motif in a more systematic way, we set out to construct a library in which a larger fraction of the possible variants of the secondary structure were represented (Fig. 1b). We previously worked out a method to construct such secondary structure libraries for functional motifs that contain canonical base pairs [25]. Because the secondary structure of Supernova contains base triples rather than base pairs, however, our strategy needed to be modified. After investigating a variety of possible options, we decided to encode each triple using the pattern YGG (where Y is C or T) or TAW (where W is A or T) (Fig. 1b). When using such a coding scheme, the probability of obtaining a base triple at a specified group of positions is 0.75 (CGG, TAA, and TAT are canonical triples but TGG is not). This approach also ensures that each of the three possible canonical triples can occur at each position in the triple helix. As in our previous method, conserved unpaired nucleotides in the secondary structure were encoded as invariant positions, and variable unpaired nucleotides were encoded as degenerate positions. Because a standard DNA synthesizer cannot be programmed to encode YGG or TAW at a specified set of positions, our library was generated by synthesizing and mixing 16 different partially degenerate oligonucleotides, each of which contained a different combination of YGG and TAW in the triple helix (Fig. 1c). This resulted in a library encoding 1.9 × 10^7^ different sequences, 1.8 × 10^6^ (9.8%) of which were consistent with the sequence requirements of the Supernova motif. To make it possible to compare this approach with standard methods, a control library was generated by random mutagenesis. This contained the same number of degenerate positions and unique sequences as the secondary structure library. However, because mutations and mutated positions were randomly chosen, only 1.5 × 10^5^ (0.78%) of the unique sequences were consistent with the secondary structure of Supernova.

**Figure 1.**
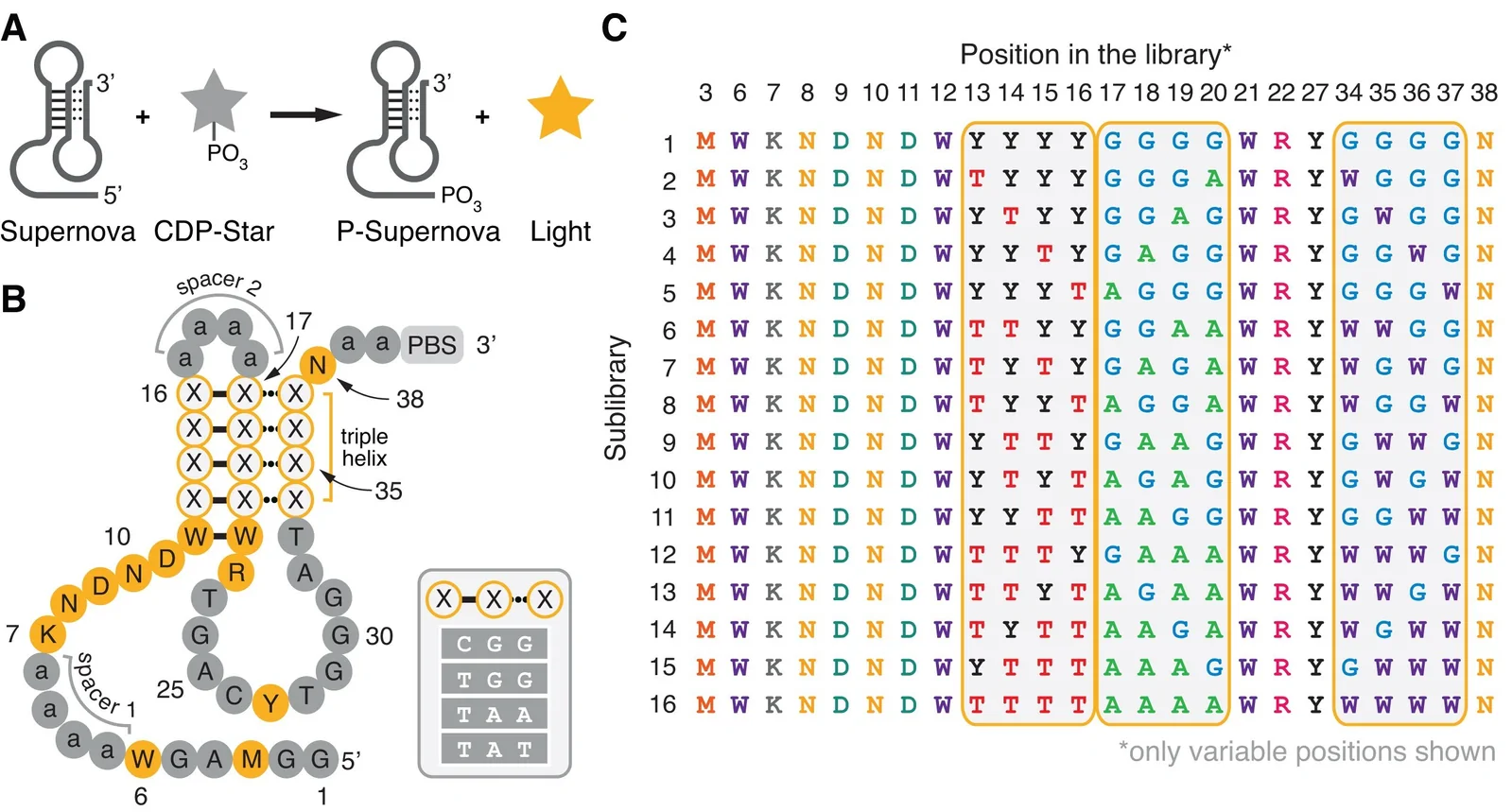
A secondary structure library based on Supernova. (**A**) Schematic representation of the reaction catalyzed by Supernova. (**B**) Design of a secondary structure library based on the Supernova motif. Purine motif base triples were encoded using groups of degenerate positions. (**C**) Sequence of the secondary structure library. This was constructed by mixing 16 different partially degenerate DNA oligonucleotides.

### Identification of Supernova variants using single-step selections

Our next objective was to identify the sequences in the secondary structure library with catalytic activity. This was achieved by first incubating under relatively stringent conditions in which Supernova (our best previously identified variant) reacted to 8%. Reacted (5′ phosphorylated) library members were then tagged by ligation, purified by PAGE, and characterized by high-throughput sequencing (Fig. 2a) [28]. Sequences were initially ranked using both CPM (counts per million) and enrichment (frequency in the evolved library divided by calculated frequency in the starting library). CPM was more strongly correlated with catalytic activity than enrichment (Fig. 2d and Supplementary Fig. S5), so we used this metric for further analysis. The evolved secondary structure library contained thousands of catalytically active sequences, including 953 with CPM values higher than that of Supernova after one round of selection (Fig. 2b). Active sequences occurred significantly more frequently among library members consistent with the Supernova motif than in those that contained at least one mismatch in the triple helix. For example, although only 4% of the 100 sequences with the highest CPM values in the starting library matched the consensus sequence of Supernova, 47% did after a single round of selection, and ~80% did after additional rounds of selection (Supplementary Fig. S6). Further analysis revealed that these variants almost never contained more than one disrupted triple (Supplementary Fig. S7), and that the disrupted triple usually occurred at positions 16-17-37 (Supplementary Fig. S8). Since previous results showed that disrupting this triple has only a small effect on activity, these results suggest that most or all of the most active variants in the library adopt the fold of Supernova. The secondary structure library also contained significantly more catalytically active sequences than the randomly mutagenized control library (Fig. 2c and Supplementary Fig. S9). Further analysis revealed that many active sequences in the secondary structure library were considerably different from previously identified variants of Supernova, although they maintained the potential to form each of the base triples in the structure (Fig. 2e). Taken together, these results show that the library contains thousands of active sequences, including some with catalytic efficiencies higher than that of Supernova. They also indicate that such variants can be rapidly identified in a single round of selection followed by high-throughput sequencing.

**Figure 2.**
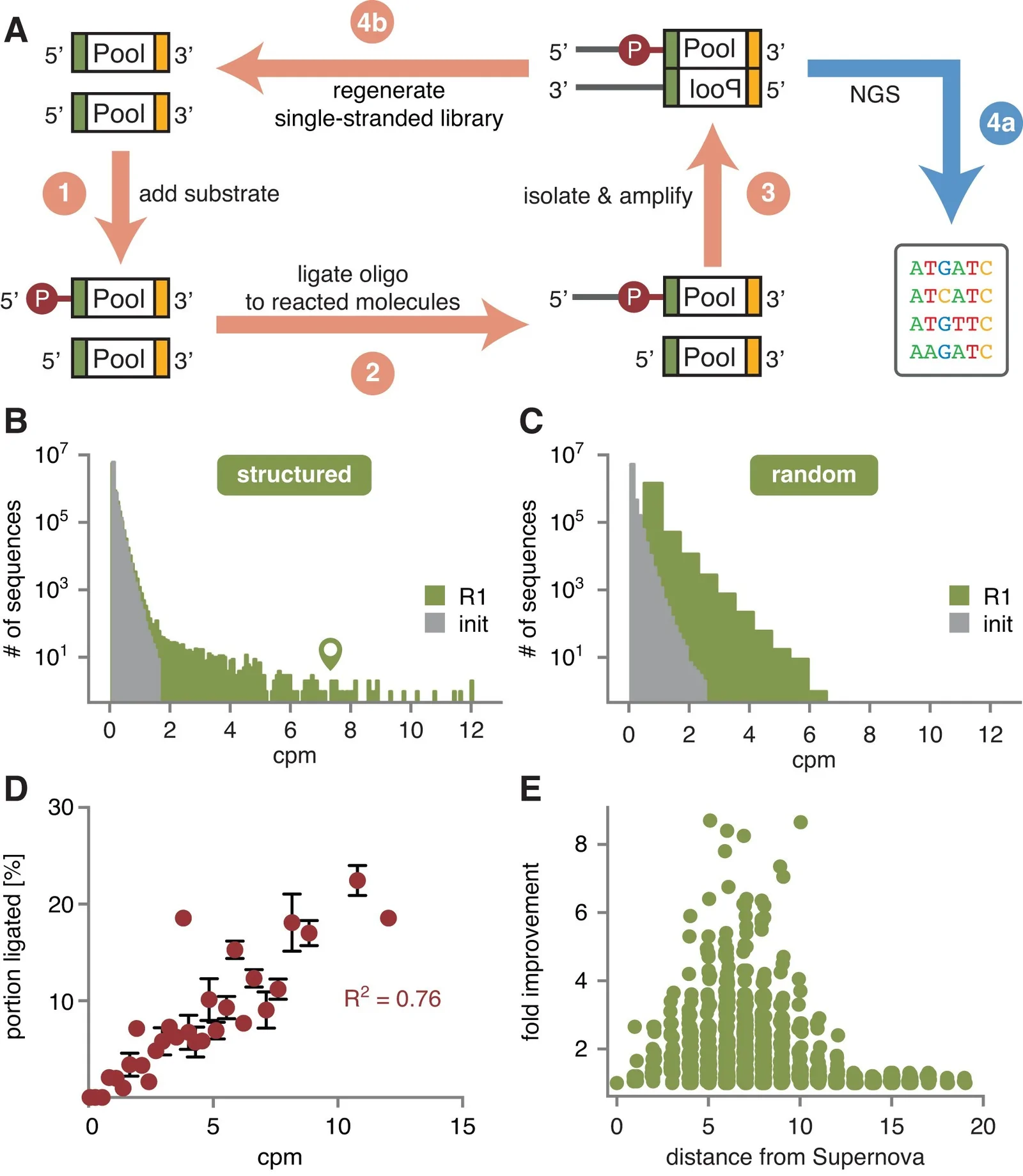
Rapid identification of Supernova variants using single-step selections. (**A**) Workflow of single and multiple-step selections. During a single-step selection, the library undergoes the incubation (1), ligation (2), and isolation and amplification (3) steps, after which it is characterized by next-generation sequencing (NGS; 4a). In a multiple-step selection, the library goes through steps 1–3, after which it is regenerated as a single-stranded library (4b). This enriched library then undergoes another step (or round; 1–3, 4b) to increase the abundance of active variants. After a sufficient number of rounds, the evolved library is characterized by NGS (4a) (**B**) Distribution of CPM values in the secondary structure library before and after selection. The position of the sequence with the highest activity after four rounds of selection is indicated (Hit 1 in Supplementary Table S1). (**C**) Same, but for a randomly mutagenized control library. (**D**) Correlation between CPM value and catalytic activity (percent ligated after a 100-second incubation with 125 μM CDP-Star) for sequences in the secondary structure library after selection. *N* = 2; error bars represent one standard deviation. (**E**) Mutational distances of sequences in the secondary structure library with CPM values greater than or equal to that of Supernova.

### Analysis of selection dynamics using high-throughput sequencing

Although the rapid discovery of improved variants of Supernova was exciting, we wondered if additional rounds would yield even more active variants. To answer this question, three additional rounds of selection were performed, and material from each round was characterized by high-throughput sequencing. By plotting the distribution of CPM values of sequences in the library after different numbers of rounds of selection, it was possible to visualize the dynamics of the selection process (Fig. 3 and Supplementary Figs S10 and S11). This showed that, as the number of rounds of selection increased from 1 to 2 and from 2 to 3, both the degree of separation between the CPM values of sequences in the starting and evolved libraries and the fraction of sequences consistent with the sequence requirements of the Supernova motif also increased (Supplementary Fig. S6). These values then reached a plateau, and did not appreciably change when another round of selection was performed. To examine these dynamics in a different way, each sequence was ranked based on its CPM value. After one round, the most active variant obtained in these selections was the 25th most abundant sequence in the library (out of ~1.9 × 10^7^ total sequences), and its CPM value of 7.3 was only 1.6-fold less than that of the most abundant sequence (Fig. 3a). After a second round of selection, the rank of this variant increased to 13, and after a third round of selection it reached a plateau of 3. In comparison, Supernova was the 1079th most abundant sequence after a single round of selection, the 239th most abundant sequence after two rounds of selection, the 111th most abundant sequence after three rounds of selection, and the 90th most abundant sequence after four rounds of selection (Fig. 3b). These results highlight the dynamics of the selection process. They also show that most of the enrichment obtained in this experiment was achieved in the first round of the selection.

**Figure 3.**
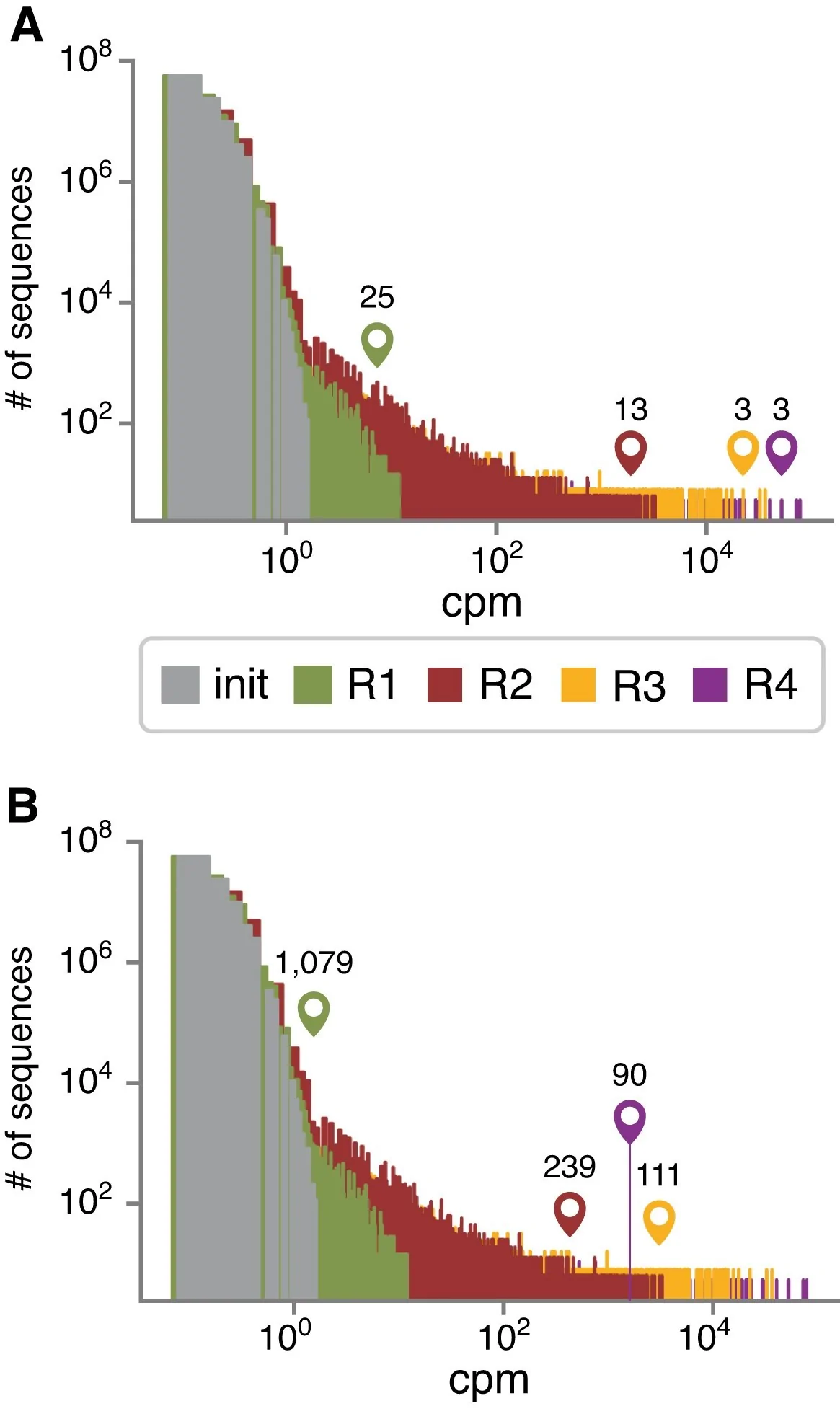
Selection dynamics. (**A**) Distribution of CPM values in the secondary structure library after 0, 1, 2, 3, and 4 rounds of selection. The rank of the sequence with the highest activity after four rounds of selection is displayed for each round (Hit 1; Supplementary Table S1). (**B**) Same as panel (A), but showing the rank of Supernova (the previously identified most active variant).

### Optimization of spacer sequences

While the secondary structure library provided extensive information about the catalytic core of the Supernova motif, it was not mutagenized in the spacer between nucleotides 6 and 7 (which was originally 26 nucleotides and replaced by the arbitrary sequence AAAA in minimization experiments) or between nucleotides 16 and 17 (which was originally 18 nucleotides and also replaced by the arbitrary sequence AAAA in minimization experiments). Previous experiments showed that variants with other spacer sequences are also catalytically active, and that mutations in these regions can even modestly increase Supernova activity. To investigate the sequence requirements of spacer and loop sequences in a more systematic way, a library was synthesized in which these two regions (referred to in this study as spacer 1 and 2) were randomized in the context of the best variant isolated from the secondary structure selection (see Supplementary Table S1; Hit 1). This library contained eight randomized positions (four in each spacer) and encoded 65 536 distinct sequences (Fig. 4a). Active variants were identified by performing a single round of selection followed by high-throughput sequencing (Fig. 4b). As before, sequences were ranked using both CPM and enrichment values. In contrast to results obtained from the secondary structure library, in this case enrichment values were a better predictor of catalytic activity than CPM values (Fig. 4c and Supplementary Fig. S12). This is likely because calculation of enrichment values requires precise knowledge of the frequencies of sequences in the starting library, and sampling of these frequencies was less complete in the secondary structure library than in the spacer library (as it encoded 288-fold more unique sequences). The 10 most abundant variants after selection were severalfold more active than any previously isolated sequences in both light-production and ligation assays (Supplementary Figs S13a and b). Further analysis of library members with the highest enrichment values after selection revealed that the sequence of spacer 1 was highly constrained among the most active variants: all were A and C rich, and the sequence CACA occurred in the top 10 variants (Fig. 4d). In contrast, each of the 10 most enriched variants of spacer 2 contained a possible base pair at positions 1 and 4 with no discernible pattern at the remaining positions (Fig. 4e and Supplementary Table S1). These results indicate that, while the arbitrary AAAA spacers previously introduced during minimization of the Supernova motif support catalytic function, optimization can yield variants with improved activity.

**Figure 4.**
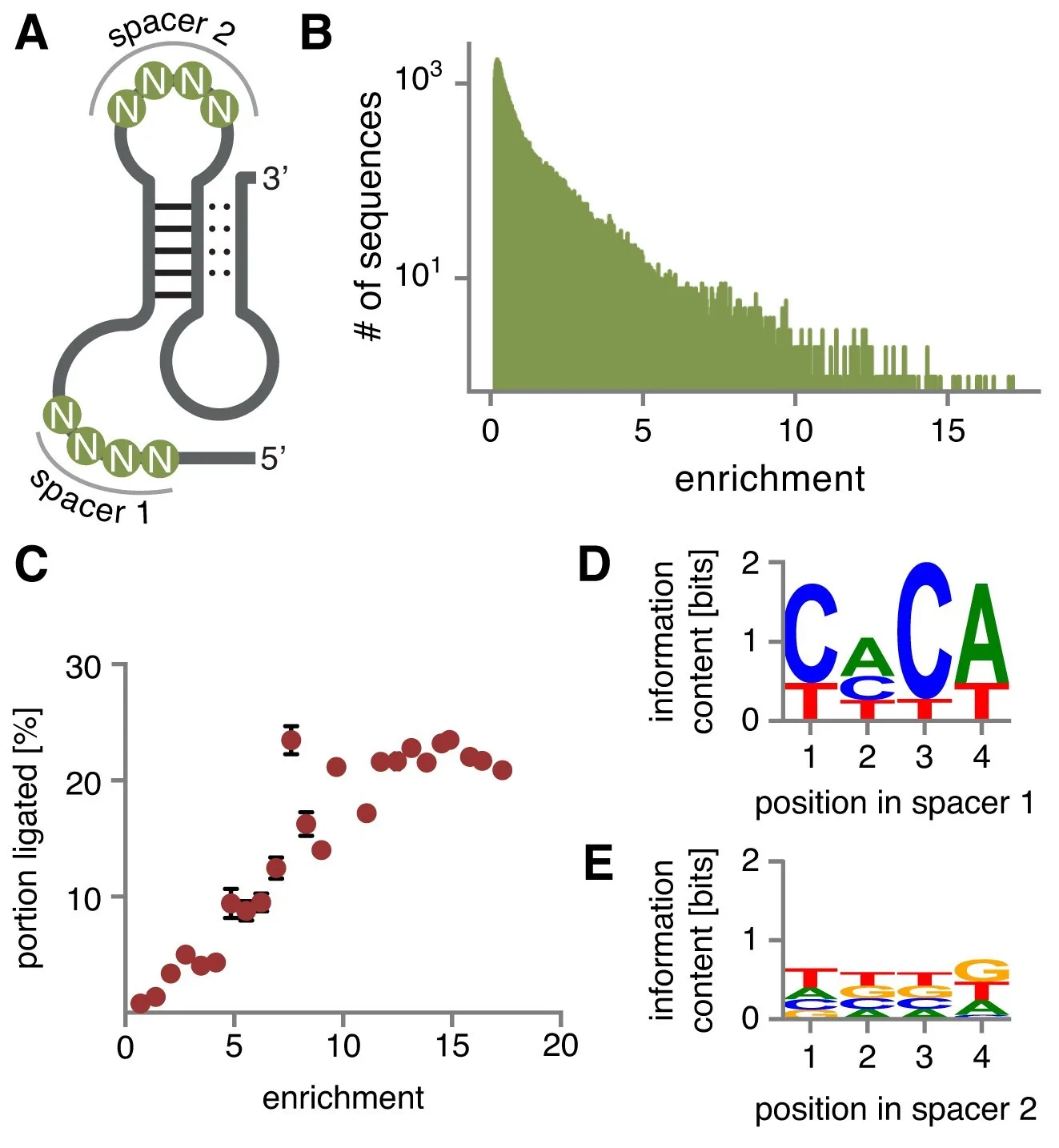
A spacer library based on Supernova. (**A**) Secondary structure of Supernova with the eight randomized positions in the spacers highlighted. (**B**) Distribution of enrichment values in the spacer library (determined by dividing the CPM value of each sequence after selection by the CPM value in the starting library). (**C**) Correlation between enrichment value and catalytic activity (percent ligated after a 100-second incubation of 1 μM deoxyribozyme with 125 μM CDP-Star in 1× selection buffer) for sequences in the spacer library after selection. *N* = 2; error bars represent one standard deviation. (**D**) Sequence logo showing conservation patterns in spacer 1. (**E**) Same, but for spacer 2.

### Combining the best cores and spacers

As a final step in the optimization workflow, the sequences of the five best variants from the secondary structure library were combined with those of the five best variants from the spacer library in each of the 25 possible combinations (Fig. 5a). These variants were tested for the ability to phosphorylate themselves in the presence of CDP-Star using a ligation assay and to produce light using a plate reader assay. Each recombinant was more active than Supernova (Fig. 5b and Supplementary Fig. S14). The catalytic rate of the most active variant, which we named Hypernova, was 97 ± 50 times higher than that of Supernova at 1 μM CDP-Star, and the maximum percent ligated was 2.2 ± 0.4 times higher (Figs. 6a–c and Supplementary Fig. S15). Its *k*_cat_ was too fast to be measured by manual pipetting, but time courses performed at 100 µM CDP-Star suggest that this value is at least 4 min^−1^ (Supplementary Fig. S16). Further characterization showed that a Cy3-labeled variant of Hypernova catalyzes self-phosphorylation with a *k*_cat_/*K*_M_ of 4 × 10^4^ ± 5.5 × 10^3^ M^−1^ min^−1^ (Fig. 6d and Supplementary Figs S17–S20). Hypernova also produced light more efficiently than Supernova, with an initial rate of light production 14 ± 3 to 30 ± 20 fold faster under the conditions we tested (Fig. 6e and Supplementary Figs S21–S23), a maximum signal to noise ratio of 13 000 (Fig. 6f) and a detection limit of 12 nM (Supplementary Fig. S24). These experiments show that by combining beneficial mutations isolated from different libraries, even more efficient variants can be obtained. They also indicate that Hypernova is significantly more active than Supernova.

**Figure 5.**
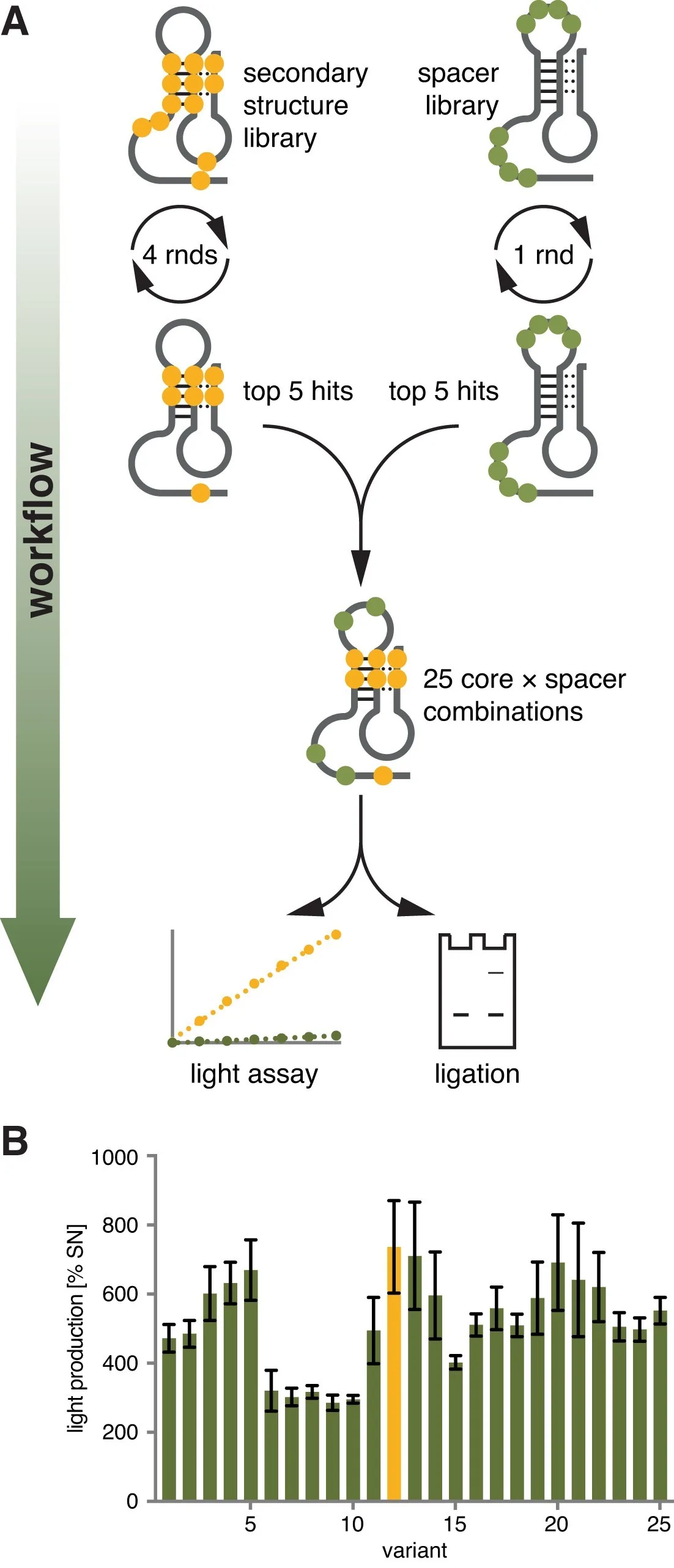
Optimization of Supernova by recombination. (**A**) Recombination workflow. (**B**) Activity of recombinants compared to Supernova as determined using a light production assay. Hypernova is indicated in orange (column number 12). Reaction conditions were 1 μM deoxyribozyme and 15 μM CDP-Star in 1× selection buffer. *N* = 3; error bars represent one standard deviation.

**Figure 6.**
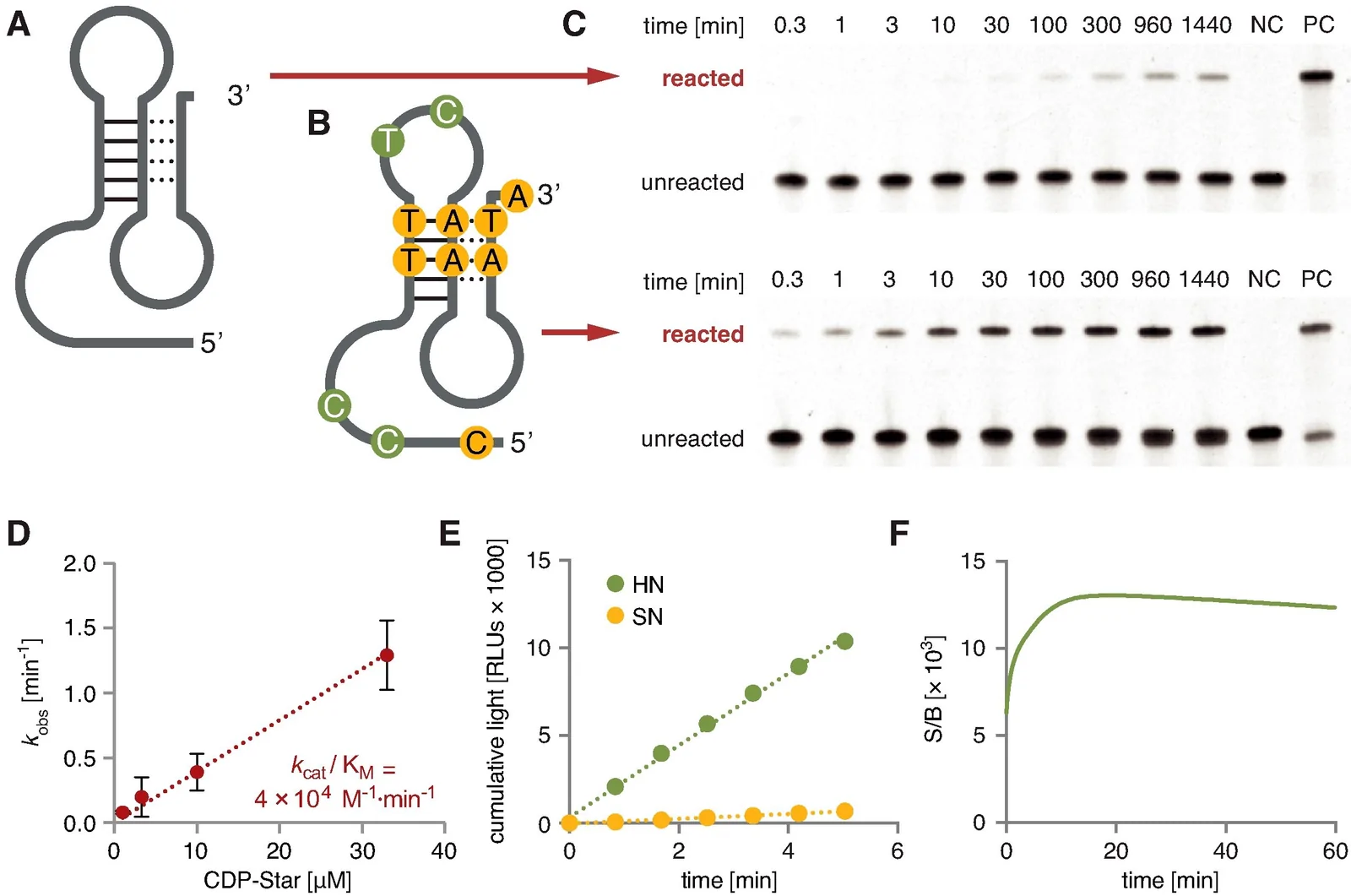
Hypernova: an optimized light-producing deoxyribozyme. (**A**) Simplified representation of the secondary structure of Supernova. (**B**) Simplified representation of the secondary structure of Hypernova with mutations highlighted. (**C**) Head-to-head comparison of the activity of Supernova and Hypernova at a subsaturating concentration of CDP-Star. Analysis was done using a ligation assay and visualized by 6% urea-PAGE. Conditions were 1 μM deoxyribozyme and 1 μM CDP-Star. (**D**) Measurement of the *k*_cat_/*K*_M_ of fluorophore-labeled Hypernova using a ligation assay. Conditions were 40 nM Hypernova and the indicated concentration of CDP-Star in 1× selection buffer. *N* = 3–5; error bars represent one standard deviation. (**E**) Representative time course showing initial rate of light production of Hypernova compared to that of Supernova. Conditions were 1 μM deoxyribozyme and 15 μM CDP-Star. (**F**) Representative time course showing the maximum signal-to-noise ratio that was achieved using Hypernova. Conditions were 30 μM deoxyribozyme and 62.5 μM CDP-Star in 1× selection buffer; S/B = signal over background.

### Enhanced performance of Hypernova sensors

To assess the utility of our improved deoxyribozymes with regard to potential biosensing applications, we compared the ability of Supernova and Hypernova to generate ligand-dependent signals in the context of three different sensing architectures previously developed in the group (Fig. 7). The first architecture can be used to detect ribonucleases that generate 2′,3′-cyclic phosphate and 5′-OH termini (Fig. 7a) [19]. Sensors consistent with this architecture were constructed by modifying the 5′-hydroxyl group of Supernova and Hypernova with a single RNA linkage. This blocks the reaction site and inactivates each deoxyribozyme. In the presence of ribonuclease, the RNA linkage is cleaved, which regenerates the 5′-hydroxyl group and restored catalytic activity. The second architecture we characterized can be used to detect DNase activity (Fig. 7c) [24]. It consists of unmodified deoxyribozyme. If DNase is present in a sample, the deoxyribozyme will be digested, which results in a reduced chemiluminescent signal. The third architecture we tested can be used to detect DNA-editing enzymes (Fig. 7e) [34]. In this case, we used a deoxyribozyme that is inactivated by the cytidine deaminase APOBEC3A, which changes C to U in single-stranded DNA and RNA and is linked to many human cancers. To facilitate interpretation of this experiment, an engineered variant of Supernova was used that contained the same number of TC motifs (the preferred editing site of APOBEC3A) as Hypernova (Supplementary Fig. S25 and Supplementary Table S1). In each of these three cases, the version of the sensor that used Hypernova as the signaling domain outperformed the version that used Supernova (or engineered Supernova in the case of the APOBEC3A sensor). This could be most clearly appreciated when comparing the initial rate of light production, which was 16-fold to 29-fold faster in Hypernova-based sensors than in sensors that used Supernova or engineered Supernova as the signaling domain (Fig. 7). However, it was also the case when signal-to-noise ratio was used as the readout. These proof of principle experiments demonstrate that sensors, which use Hypernova perform significantly better than those which use Supernova in a wide range of assays.

**Figure 7.**
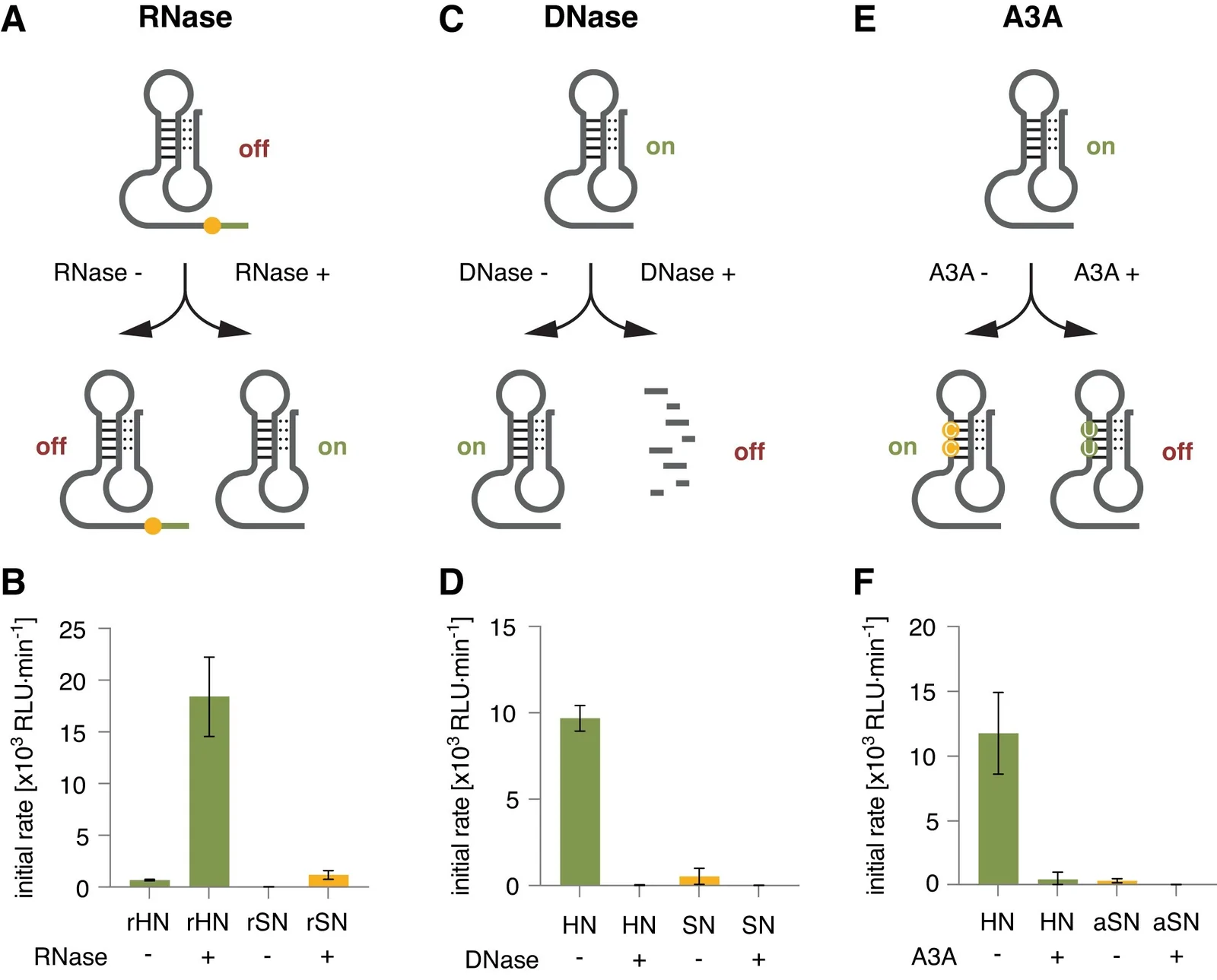
Enhanced performance of Hypernova sensors. (**A**) RNase sensor. (**B**) Initial rates of light production of sensors that use either Hypernova (rHN) or Supernova (rSN) as a signaling domain. Sensors were tested after preincubation in either the absence or presence of RNase A. (**C**) DNase sensor. (**D**) Initial rates of light production of sensors that use either Hypernova (HN) or Supernova (SN) as a signaling domain. Sensors were tested after preincubation in either the absence or presence of DNase I. (**E**) APOBEC3A sensor. (**F**) Initial rates of light production of sensors that use either Hypernova (HN) or Supernova (aSN) as a signaling domain. Sensors were tested after preincubation in either the absence or presence of APOBEC3A. In all cases, *N* = 3, and error bars represent one standard deviation.

## Discussion

Nucleic acids with a desired function can often be isolated from random sequence libraries, but typically require optimization before they are useful for practical applications. A common strategy to achieve this goal is to make a library by randomly mutagenizing the initial isolate of a motif at a low level, and identify the active variants in a second selection experiment. In many cases, functional molecules can benefit from further optimization, but the search for better variants tends to become progressively more difficult as a motif is improved due to the shape of the fitness distribution. Here, we addressed this challenge in the context of a previously optimized light-producing deoxyribozyme called Supernova. In one approach, a library was constructed in such a way that ~10% of possible variants were consistent with the secondary structure and sequence requirements of the Supernova motif (Figs 1 and 2). Care was taken to minimize the probability of disrupting base triples while ensuring that the canonical triples CGG, TAA, and TAT could occur at each position in the triple helix. In a second approach, two AAAA spacers that were inserted during previous minimization experiments were randomized in the context of the best variant isolated from the secondary structure library (Fig. 4). Both of these libraries yielded improved deoxyribozymes. We also tested 25 recombinants in which mutations from the best variants from these two libraries were combined (Fig. 5). While effects of beneficial mutations were almost never independent, recombinants were often more active than either of the parental sequences. The sequence of our best variant, called Hypernova, contained 12 mutations relative to Supernova (Fig. 6). Since a randomly mutagenized library based on Supernova would only contain a tiny fraction of the 2.1 × 10^16^ possible sequences at this mutational distance (Supplementary Fig. S26) [35], it is unlikely that Hypernova could have been identified using random mutagenesis alone. These experiments show how, once the secondary structure and sequence requirements of a motif are known, its sequence space can be searched in a more focused way for rare variants with improved activities.

Our results also provide insights into important technical aspects of single-step selection experiments. One question is whether a single round of selection can be sufficient. In general, this depends in a complicated way on the size of the library, the amount of enrichment obtained in the selection step, and the number of reads obtained by high-throughput sequencing (selections that use smaller libraries, utilize more powerful methods of separation, and are analyzed more comprehensively by high-throughput sequencing typically require fewer rounds of selection than those that do not). It also depends on the method of analysis: counting individual sequences (which is what was done here and in previous studies in our group [28]) or counting enriched k-mers in selected sequences (which can make it possible to identify functional DNA and RNA molecules in libraries in which the ratio of reads to library members is low [36], but only works for relatively contiguous motifs that are not inhibited by flanking sequence [37]). In the case of the secondary structure library used here, results did not significantly change when additional rounds of selection were performed (Fig. 3). This indicates that, when using libraries of ~10^7^ sequences, a sequencing method that yields ~10^7^ reads, and a simple purification like PAGE to isolate active sequences, single-step selections can be used to rapidly identify the most active sequences in the library. Our experiments also provide insights into the best way to rank sequences after single-step selections. In the case of the secondary structure library, CPM values were more strongly correlated with activity than enrichment values, whereas enrichment values gave better results in the case of the spacer library. This discrepancy is probably related to differences in sequencing coverage, and the optimal metric for ranking sequences appears to depend on the ratio of reads to sequences in the starting library. Based on these results, we suggest that establishing a correlation between CPM (or enrichment) values and catalytic activity should be thought of as an important step in the workflow of single-step selection experiments.

The catalytic proficiency of Hypernova was assessed using several different approaches (Table 1). Initial characterization showed that Hypernova promotes self-phosphorylation with a rate of 0.16 ± 0.05 min^−1^ at 1 μM CDP-Star and 1 μM deoxyribozyme, while the rate of Supernova is 97-fold slower (0.0017 ± 0.0008) under these conditions. Further characterization showed that the *k*_cat_/*K*_M_ of Cy3-labeled Hypernova is 4 × 10^4^ ± 5.5 × 10^3^ M^−1^ min^−1^. This is 35-fold higher than the previously determined *k*_cat_/*K*_M_ of Supernova [17]. It is also higher than ribozyme and deoxyribozyme motifs that use nucleotide triphosphates as phosphate donors [38–40], and comparable to the most efficient artificial ribozymes that use small molecule substrates, such as a self-aminoacylating ribozyme with a *k*_cat_/*K*_M_ of 6 × 10^4^ M^−1^ min^−1^ [41] and a Diels–Alderase ribozyme with a *k*_cat_/*K*_M_ of 1.3 × 10^5^ M^−1^ min^−1^ [42]. Hypernova also generated light more efficiently than Supernova, with an initial rate of light production 14 to 30-fold faster and a maximum signal to noise ratio of more than 13 000. This signal to noise ratio is similar to those of RNA aptamers that generate fluorescent signals. [11, 12, 43, 44]. However, an advantage of Hypernova for some applications is that it is made of DNA, which means that it is less expensive to manufacture and significantly more stable. These characteristics are particularly desirable in the context of deoxyribozyme sensors for applications such as diagnostics and high-throughput screening. In this regard, we previously showed that the Supernova motif can be used to detect oligonucleotides [17] and DNA editing enzymes [34], while another group demonstrated that it can be used to detect the activity of CRISPR–Cas9 [45]. We also recently developed fluorescent and colorimetric deoxyribozyme sensors that can detect oligonucleotides, RNases, and DNases [19, 24]. Our sensors do not require washes or purifications, detect ligands with detection limits typically ranging from 100 nM to 100 pM, and can be used to identify inhibitors in high-throughput screens. They also perform better when Hypernova rather than Supernova is used as the signaling domain (Fig. 7). We anticipate that the improved activity of Hypernova will significantly increase the utility of light-producing deoxyribozymes for such applications.

**Table 1.** Comparing the performance of Supernova and Hypernova

	*k*_obs_ at 1 μM CDP-Star [min^−1^]	*k*_cat_/*K*_M_ [M^−1^⋅min^−1^]	Relative initial rate of light production
Hypernova	0.16 ± 0.05	40 000 ± 5 500	30 ± 20
Supernova	0.0017 ± 0.0008	1150	1

The *k*_cat_/*K*_M_ value of Supernova is from reference 17. The initial rate of light production was measured at 1 μM deoxyribozyme and 15 μM CDP-Star, and calculated from the first 5 min of the reaction.

## Data availability

Raw data, processed data, processing scripts and analysis notebooks are available at https://doi.org/10.6084/m9.figshare.34097340.

## Supplementary Material

gkag1143_Supplemental_File
