# Supplementary material for: Hypernova: a faster and brighter light-producing deoxyribozyme
Source: Nucleic Acids Res. 2026 Nov 13;54(21):gkag1143. doi: 10.1093/nar/gkag1143 (PMC13760218; doi:10.1093/nar/gkag1143)
Supplement: Supplementary file 1 [file gkag1143_supplemental_file.pdf]

SUPPLEMENTARY INFORMATION

Hypernova: a faster and brighter light-producing deoxyribozyme

Katerina Svehlova[1], Jaroslav Kurfürst[1,2], Martin Jakubec[1,3], and Edward A. Curtis[1*]

[1]Institute of Organic Chemistry and Biochemistry of the Czech Academy of Sciences, Prague 166 10, Czech Republic

[2]Department of Informatics and Chemistry, University of Chemistry and Technology, Prague 166 28, Czech Republic

[3]Department of Genetics and Microbiology, Faculty of Science, Charles University in Prague, Prague 128 44, Czech Republic

Phone: +420 733 169 654
E-mail: curtis@uochb.cas.cz

*Corresponding author

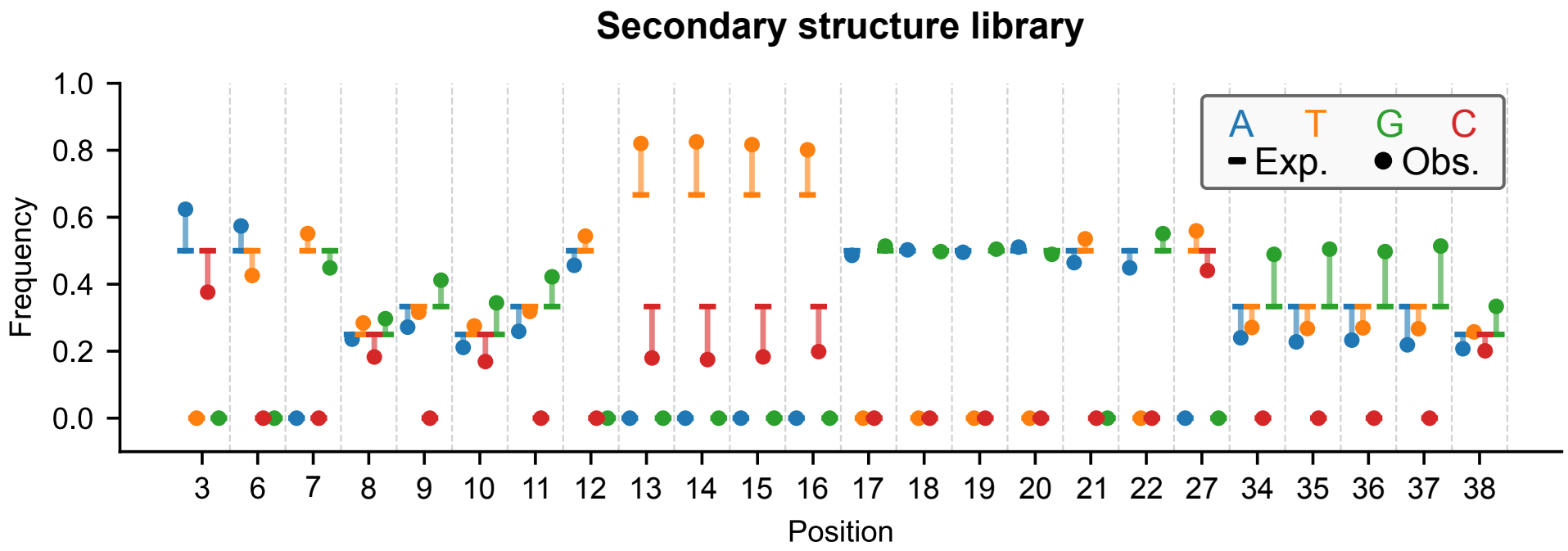

Supplementary Figure S1. Expected and observed frequencies at variable positions in the secondary structure library before selection.

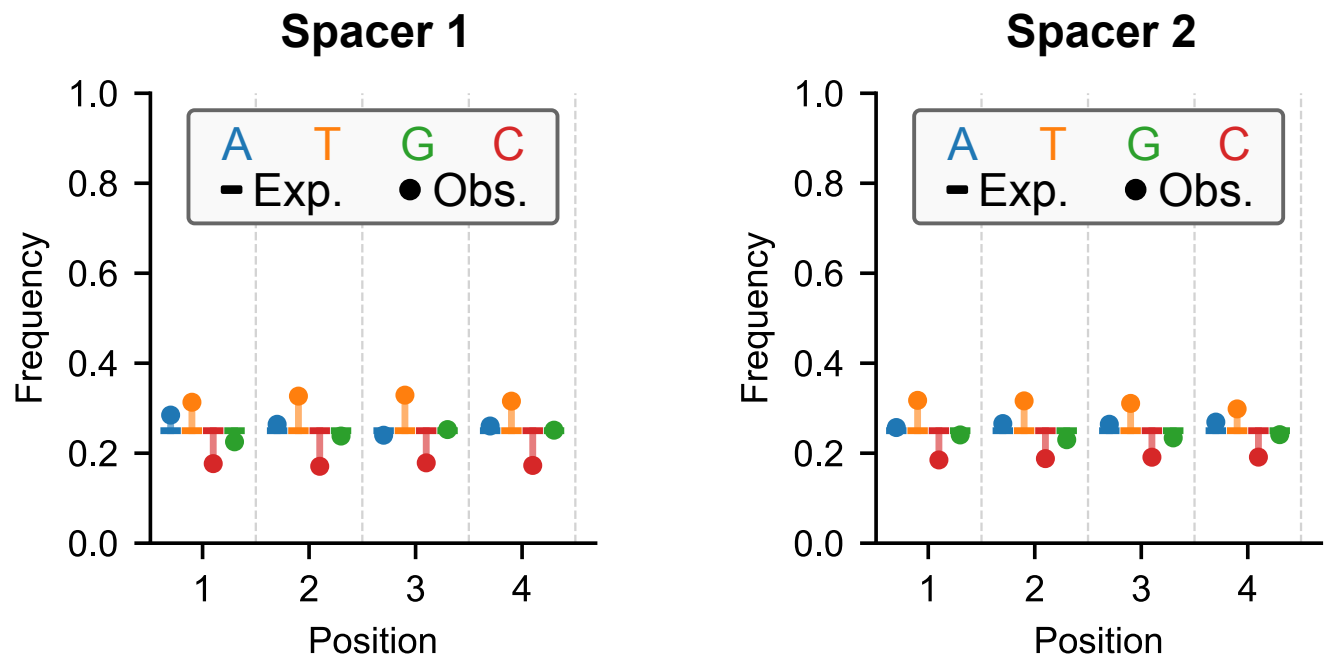

Supplementary Figure S2. Expected and observed frequencies at variable positions in the spacer library before selection.

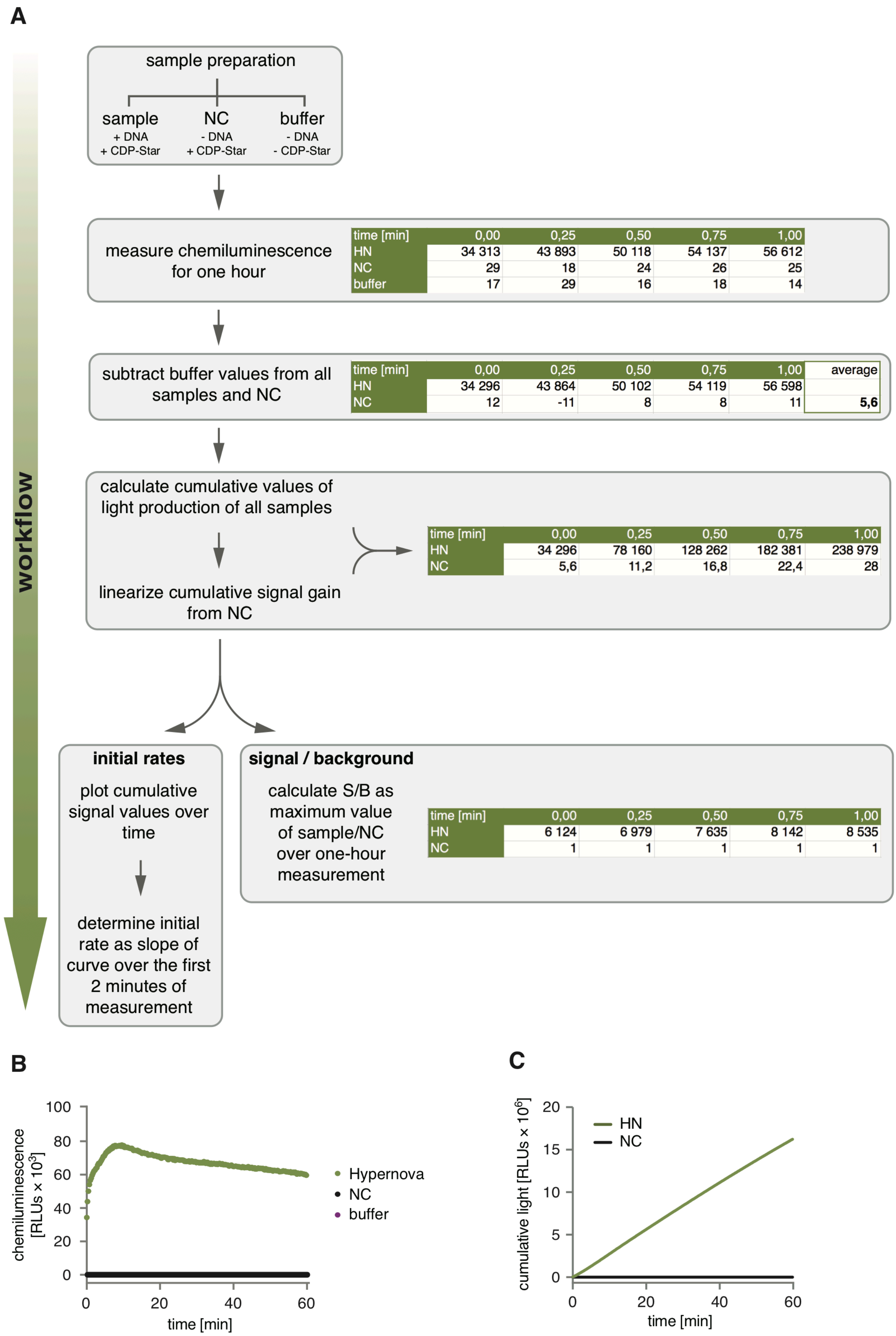

Supplementary Figure S3. Calculation of signal-to-background ratios and initial rates of light production. A) Workflow of data analysis from the light production assay. Each measurement of light production included deoxyribozyme samples (deoxyribozyme and CDP-Star in 1× selection buffer) a negative control (CDP-Star in 1× selection buffer) and a buffer sample (1× selection buffer). After 1 hour of continuous light production measurements, the data were analyzed in the following way. First, the buffer value was subtracted from the value of each sample at the respective time point. For all samples except the NC

(negative control), the cumulative light production was calculated by adding up the resulting values (from which buffer values had been subtracted) at the corresponding time points. Next, the average signal per measurement of the NC was calculated over the 60-minute period. This value was used to calculate the background cumulative light production over the complete time course. Finally, we divided the cumulative light value of each sample at each time point by the cumulative value of the NC. The final signal-over-background ratio is the maximum value over the 60-minute experiment. Alternatively, to determine the initial rates of light production, the last step is to plot the first 2 minutes of cumulative light production of each sample and the NC. These curves were fitted by linear regression and the slope was calculated to determine the initial rate of light production. B) A representative measurement of the chemiluminescent signal of Hypernova, negative control, and background over a 60-minute time course. Note that the buffer signal is at the same place as the NC at this scale. Reaction conditions were 30 μM deoxyribozyme and 62.5 μM CDP-Star in 1× selection buffer. RLU = relative luminescence units. C) The same measurement as B) after data processing. The plot shows cumulative light production over a 60-minute time course.

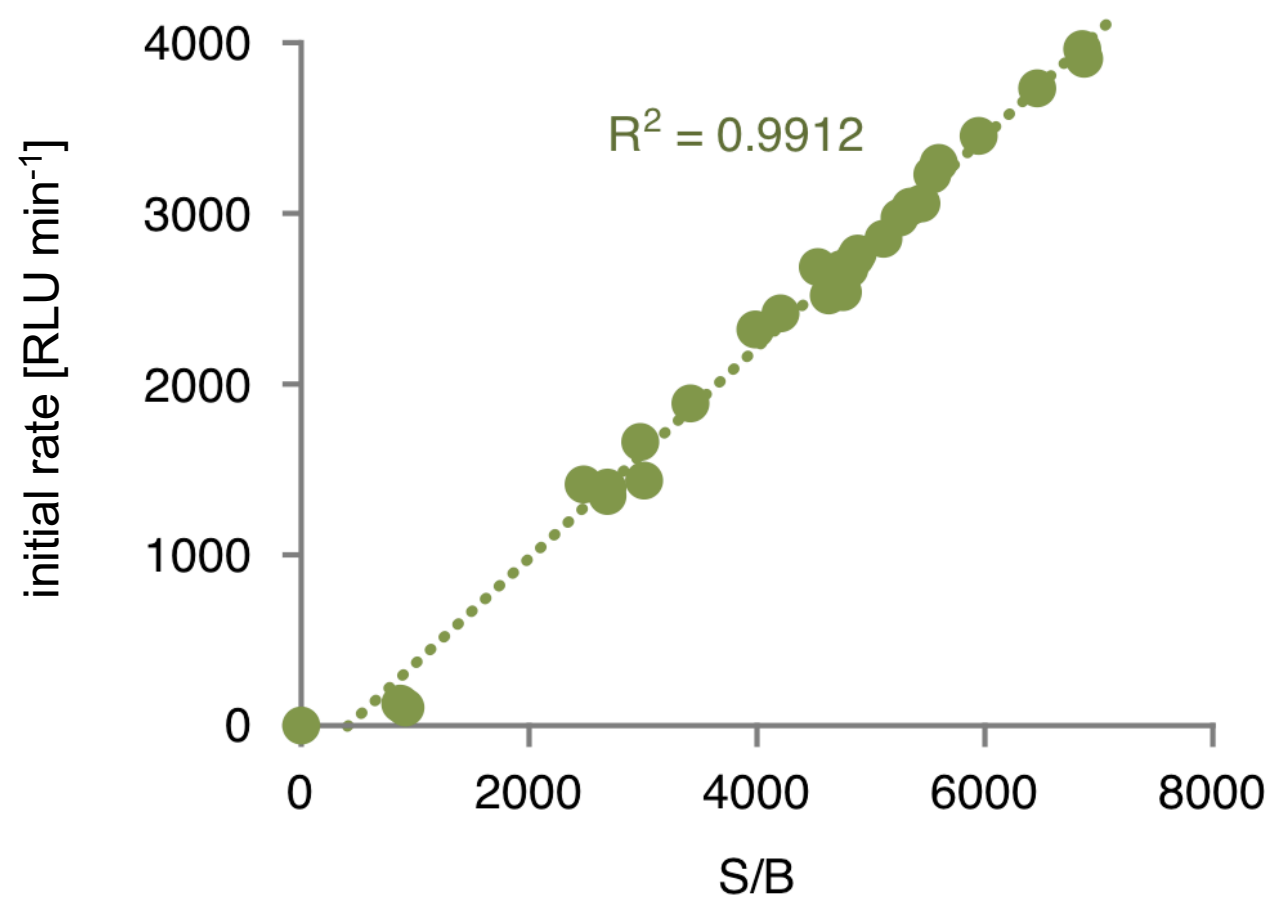

Supplementary Figure S4. Comparison of initial rates and signal-over-background values. Supernova and 25 Hypernova variants (see also Figure 5B) were tested using the light production assay and the results were analyzed to determine initial rates and signal-over-background (S/B) values for each sample. The values were linearly correlated with a goodness of fit $R^2 = 0.9912$. initial rate [RLU min^{-1}]

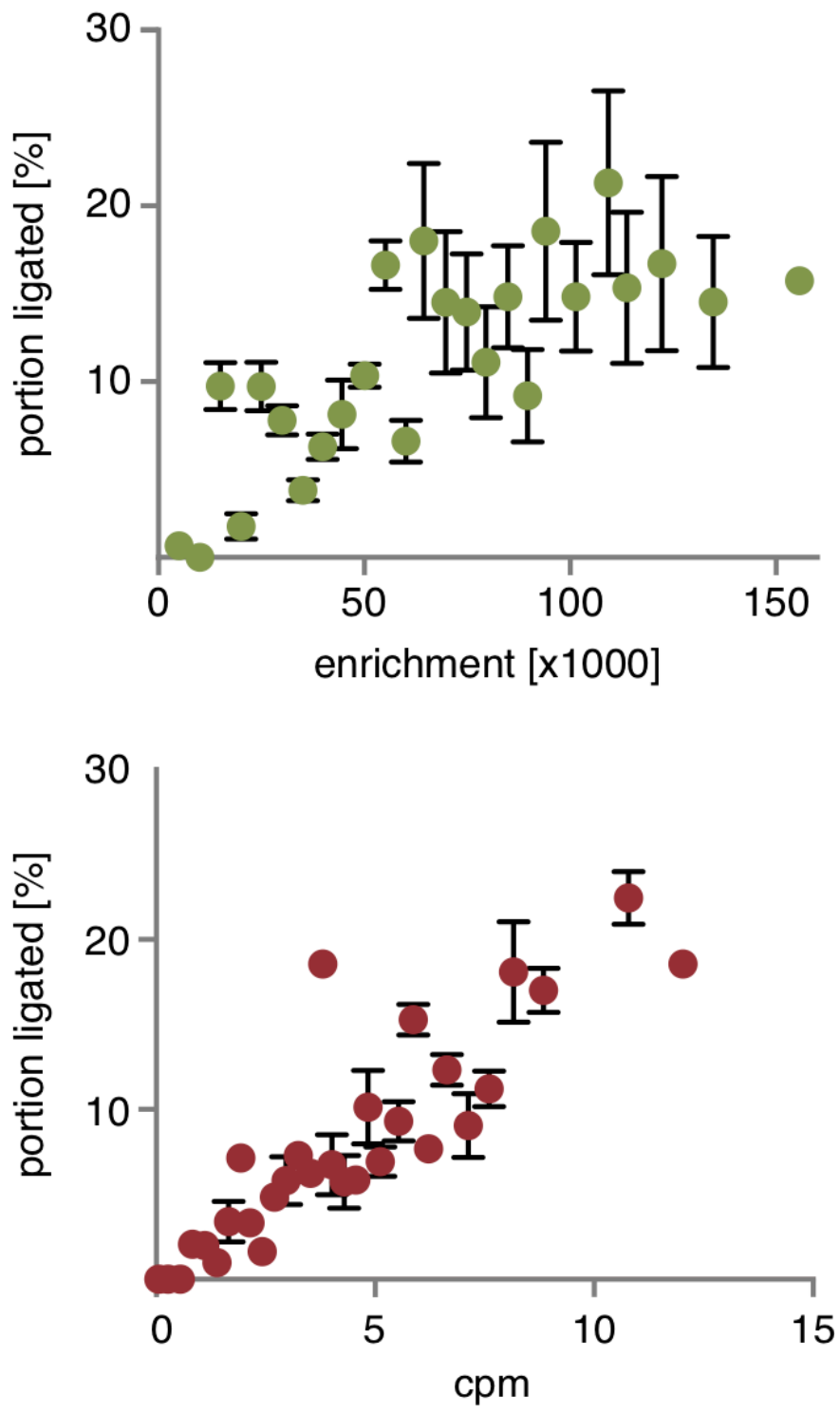

Supplementary Figure S5. Above: correlation between enrichment value and catalytic activity for sequences in the secondary structure library after one round of selection. Below: correlation between CPM value and catalytic activity for the same sequences. Reaction conditions were 1 µM deoxyribozyme and 125 µM CDP-Star in 1× selection buffer. N = 2; error bars represent standard deviation.

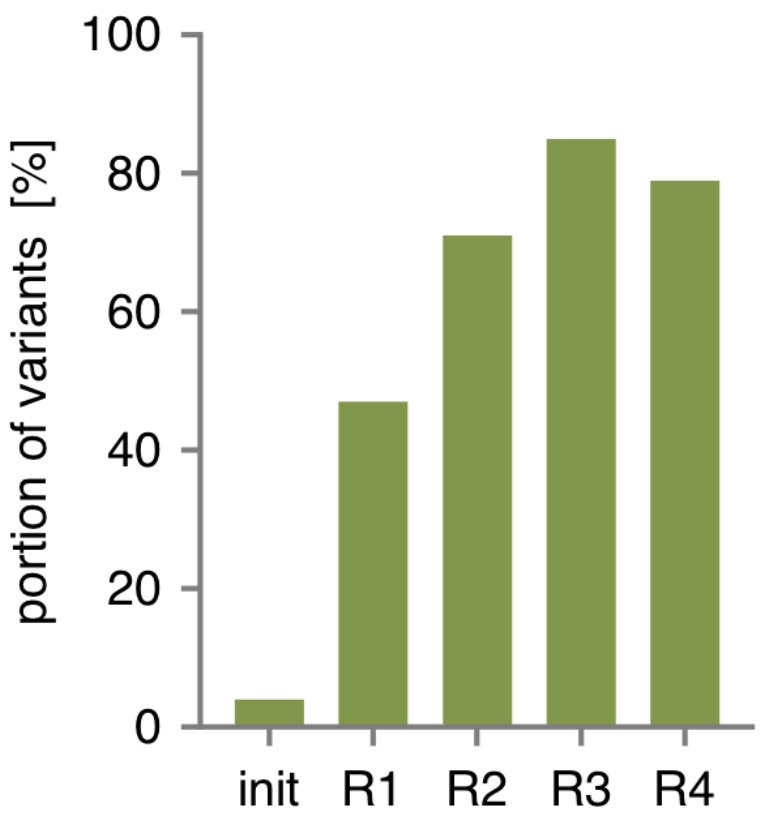

Supplementary Figure S6. Percentage of variants in the top 100 hits from the secondary structure library that match the consensus sequence of Supernova. The sequences shown correspond to the initial library (init) and rounds 1 - 4 (R1 - R4).

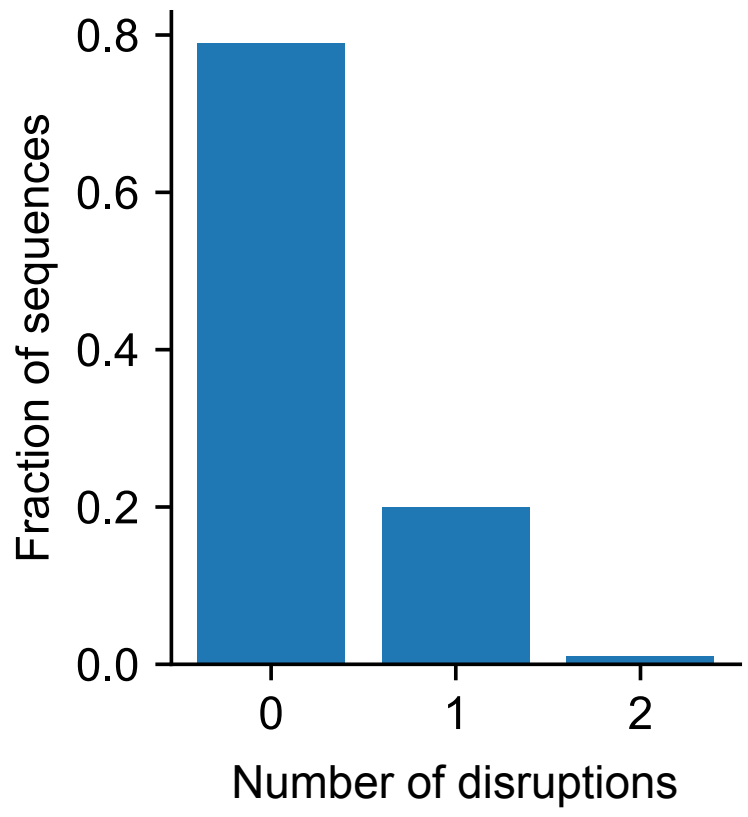

Supplementary Figure S7. Percentage of variants in the top 100 hits from the secondary structure library with different numbers of mismatches in the triple helix.

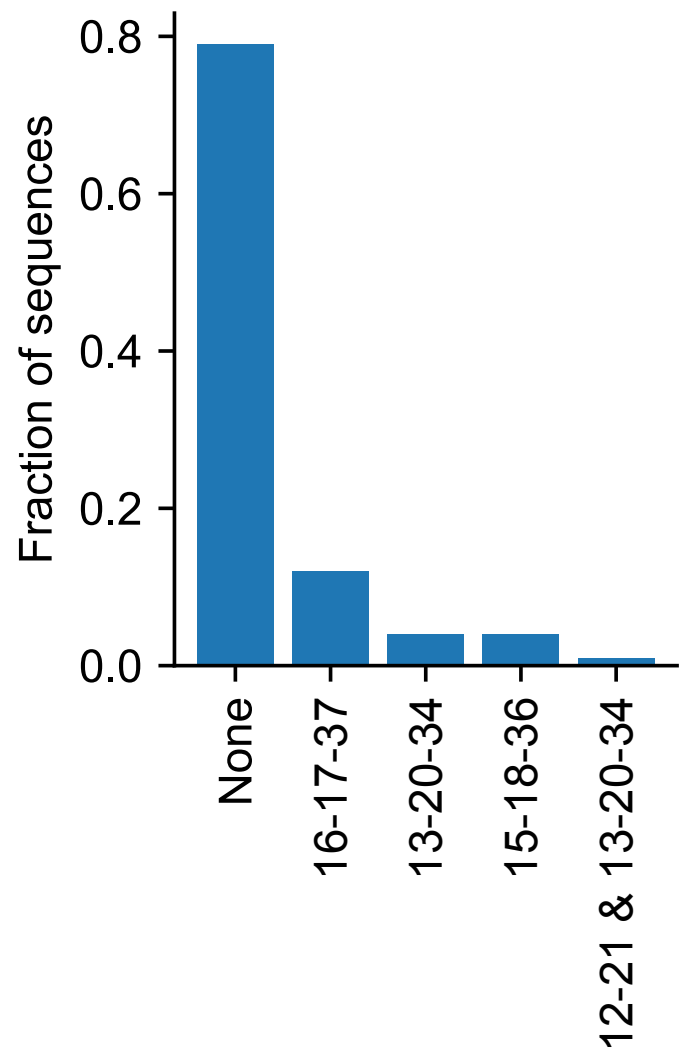

Supplementary Figure S8. Location of mismatches in variants in the top 100 hits from the secondary structure library.

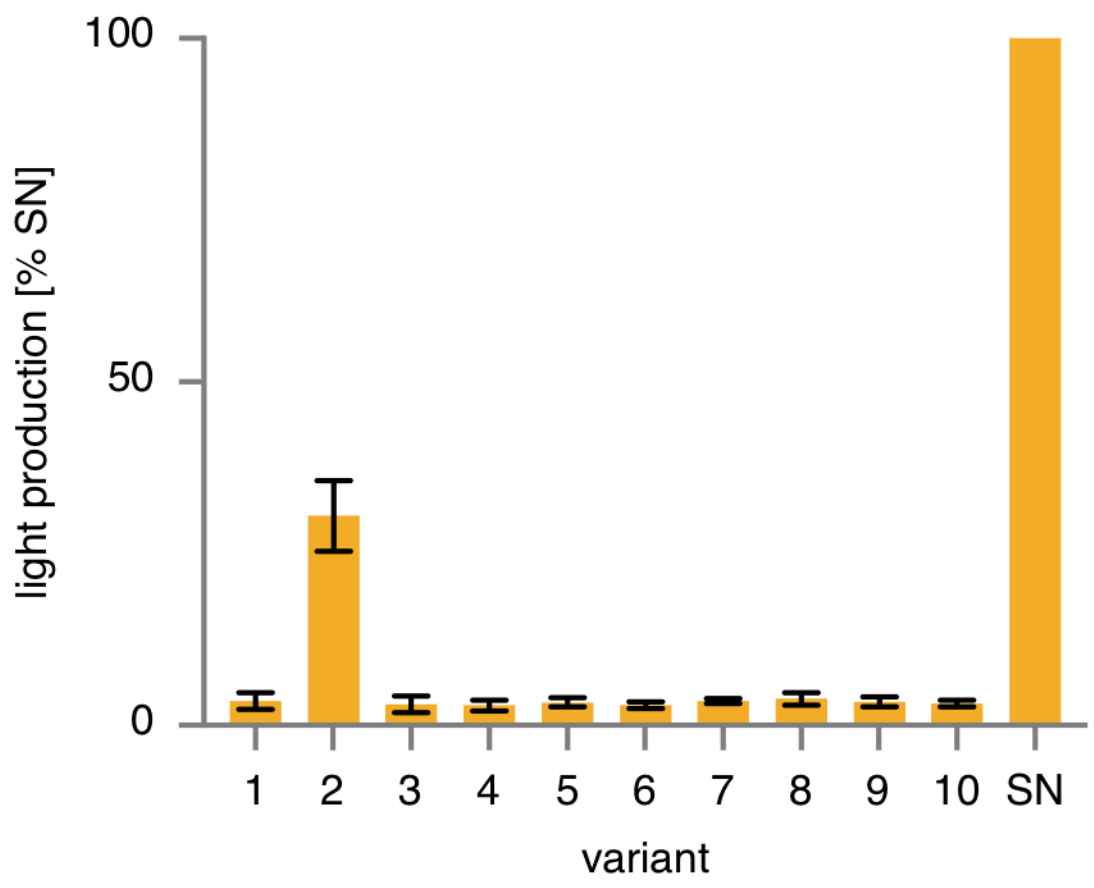

Supplementary Figure S9. Light production of the top ten variants from the randomly mutagenized control library after one round of selection. Reaction conditions were 1 μM deoxyribozyme and 125 μM CDP-Star in 1× selection buffer. SN = Supernova. Light production corresponds to the highest signal to noise ratio obtained in a 60-minute reaction and is expressed relative to that of Supernova. N = 3; error bars represent standard deviation.

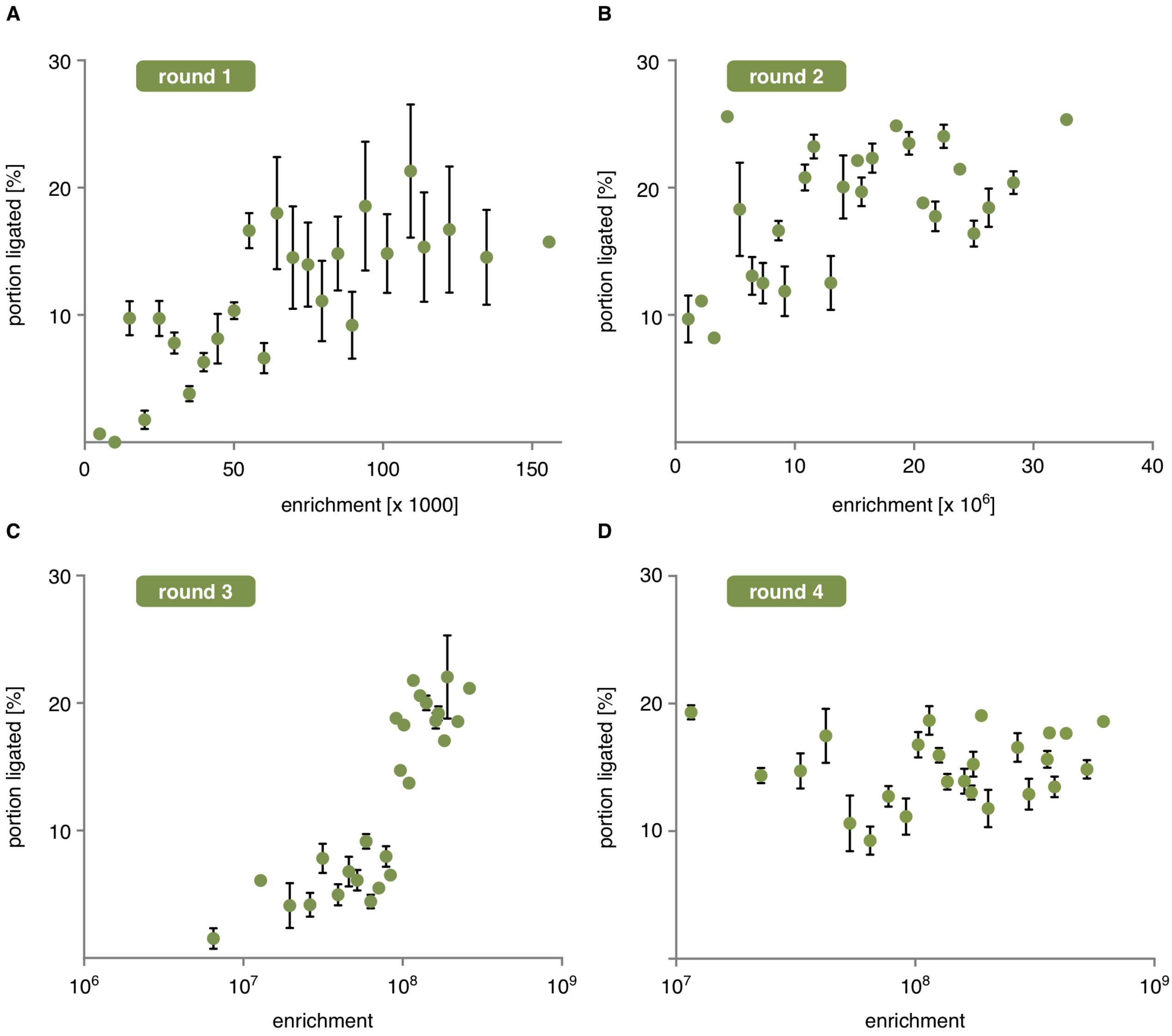

Supplementary Figure S10. Correlation between enrichment value and catalytic activity for sequences in the secondary structure library after each round of selection. Reaction conditions were 1 μM deoxyribozyme, 125 μM CDP-Star, and 1× selection buffer. After incubating for 100 seconds, activity was measured using the ligation assay. N = 2; error bars represent standard deviation.

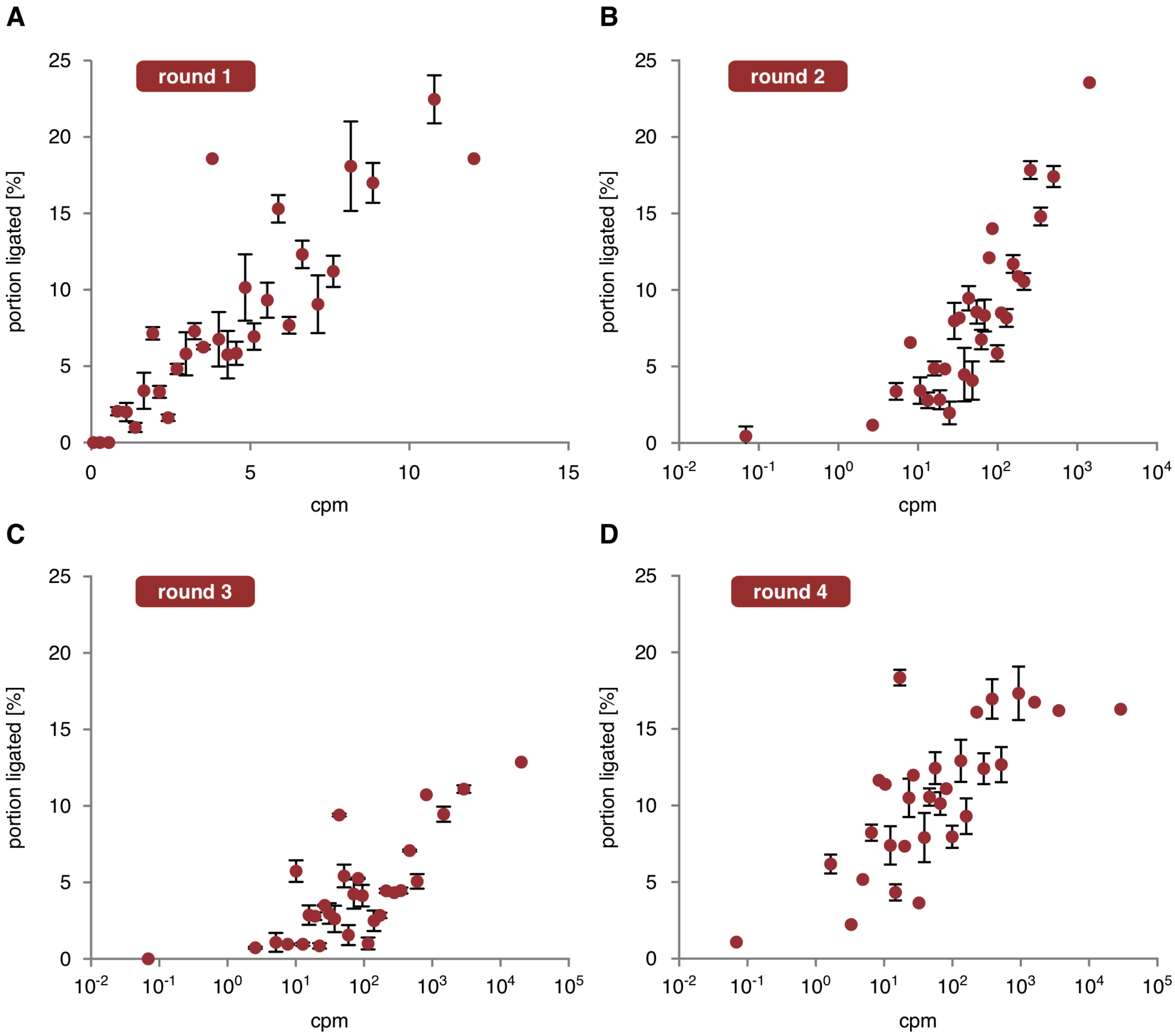

Supplementary Figure S11. Correlation between CPM value and catalytic activity for sequences in the secondary structure library after each round of selection. Reaction conditions were 1 µM deoxyribozyme, 125 µM CDP-Star, and 1× selection buffer. After incubating for 100 seconds, activity was measured using the ligation assay. In all cases n = 2; error bars represent standard deviation.

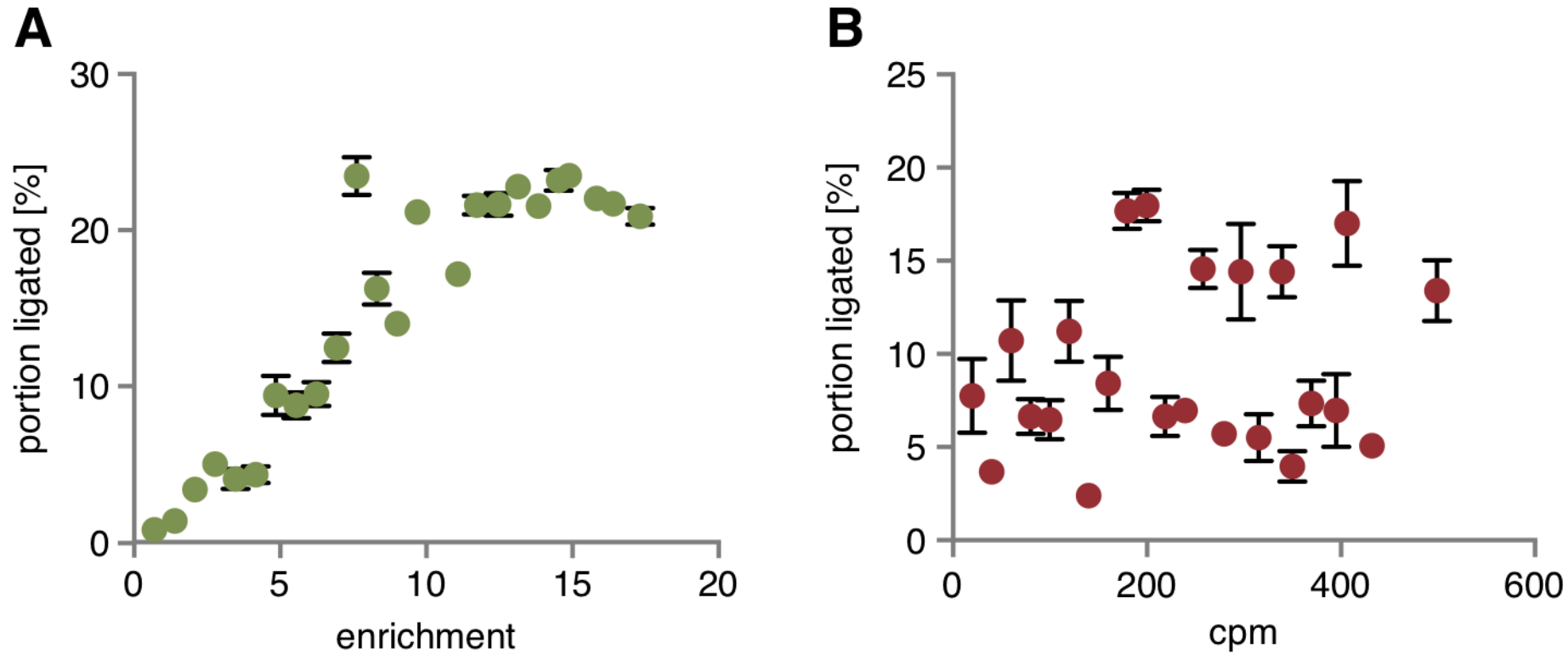

Supplementary Figure S12. A) Correlation between enrichment value and catalytic activity for sequences in the spacer library after one round of selection. B) Correlation between CPM value and catalytic activity for the same sequences. Reaction conditions were 1 µM deoxyribozyme, 15 µM CDP-Star, and 1× selection buffer. After incubating for 10 seconds, activity was measured using the ligation assay. In both cases n = 2; error bars represent standard deviation.

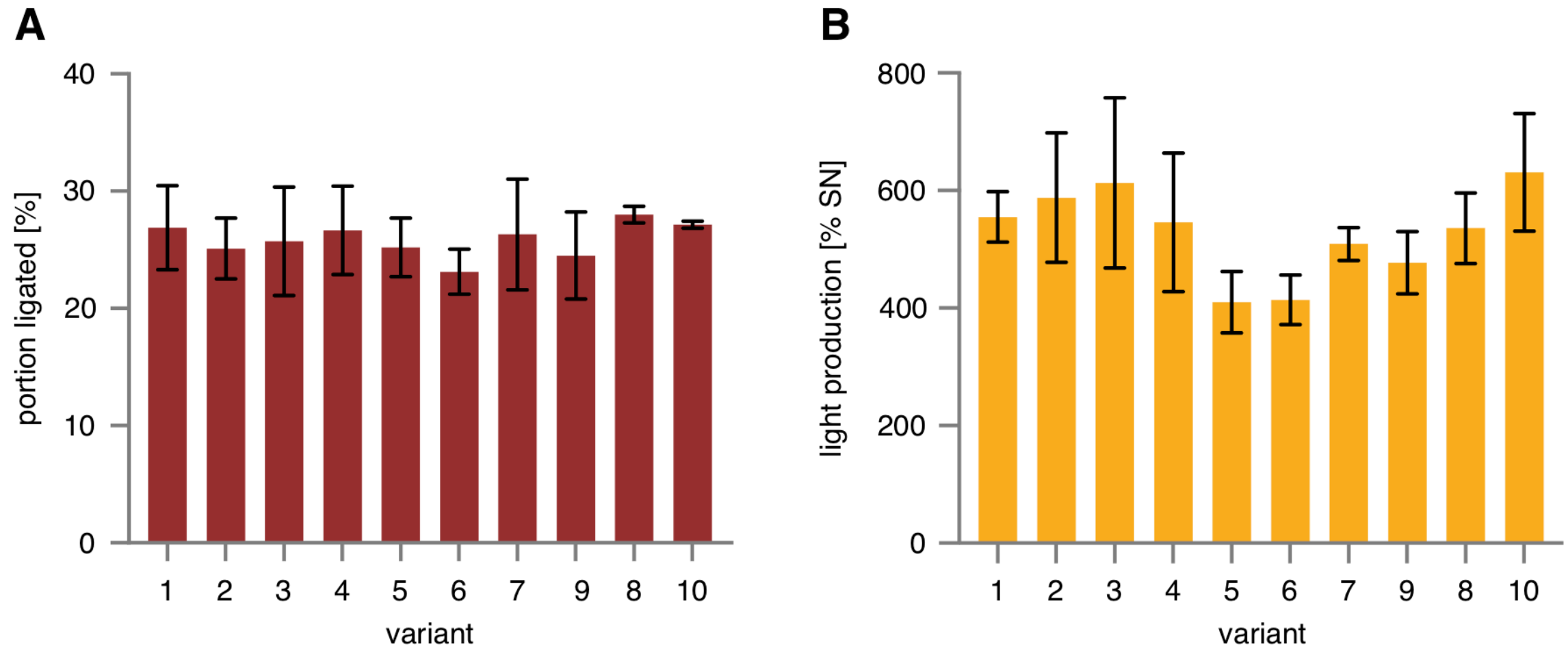

Supplementary Figure S13. Top 10 variants from the spacer library selection. A) Activities of the top 10 variants tested using the ligation assay. Reactions were analyzed after a 10-second incubation. N = 2; error bars represent standard deviation. B) Activities of the top 10 variants tested using the light production assay. Light production corresponds to the highest signal to noise ratio obtained in a 60-minute reaction, and is expressed relative to that of Supernova. N = 3; error bars represent standard deviation. Reaction conditions in both assays were 1 µM deoxyribozyme and 15 µM CDP-Star in 1× selection buffer.

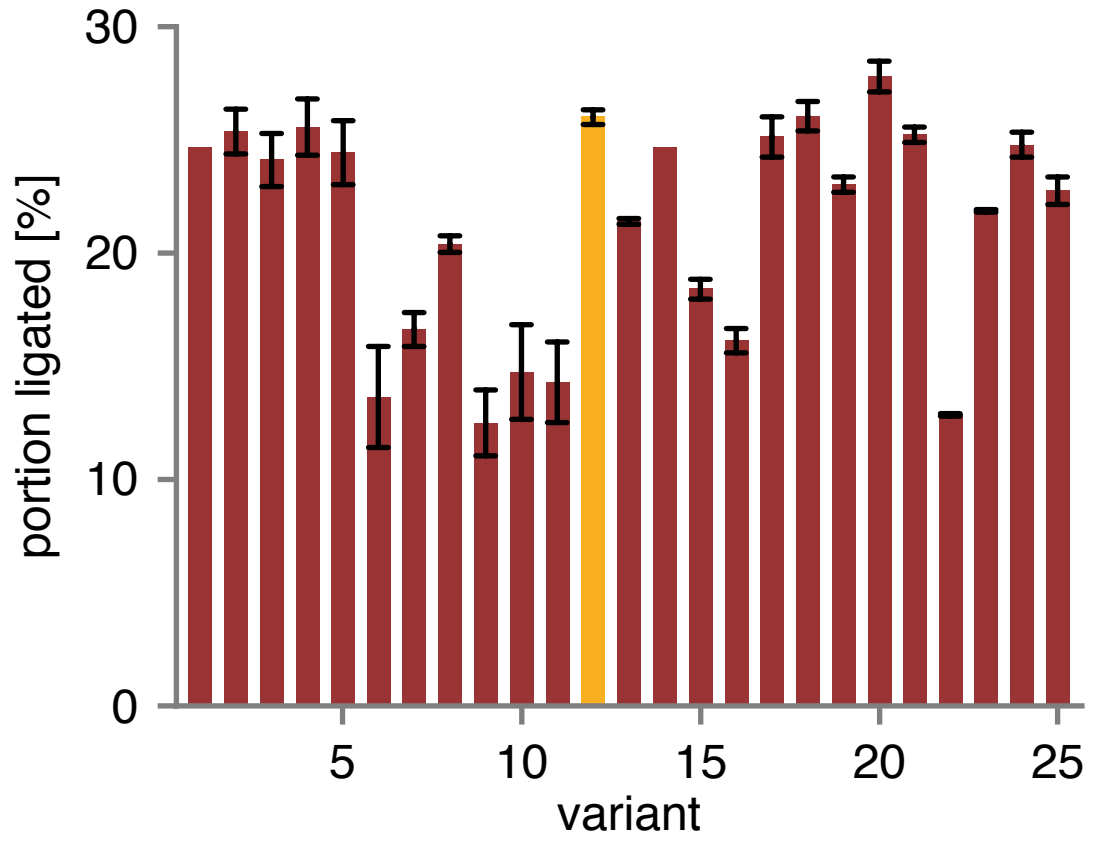

Supplementary Figure S14. Activity of recombinants in which mutations from the best 5 variants from the secondary structure library were combined with mutations from the best 5 variants from the spacer library. Hypernova sequence is indicated in yellow (column number 12). Reaction conditions were 1 μM deoxyribozyme and 15 μM CDP-Star incubated in 1× selection buffer. After incubating for 10 seconds, activity was measured using the ligation assay. N = 2; error bars represent standard deviation.

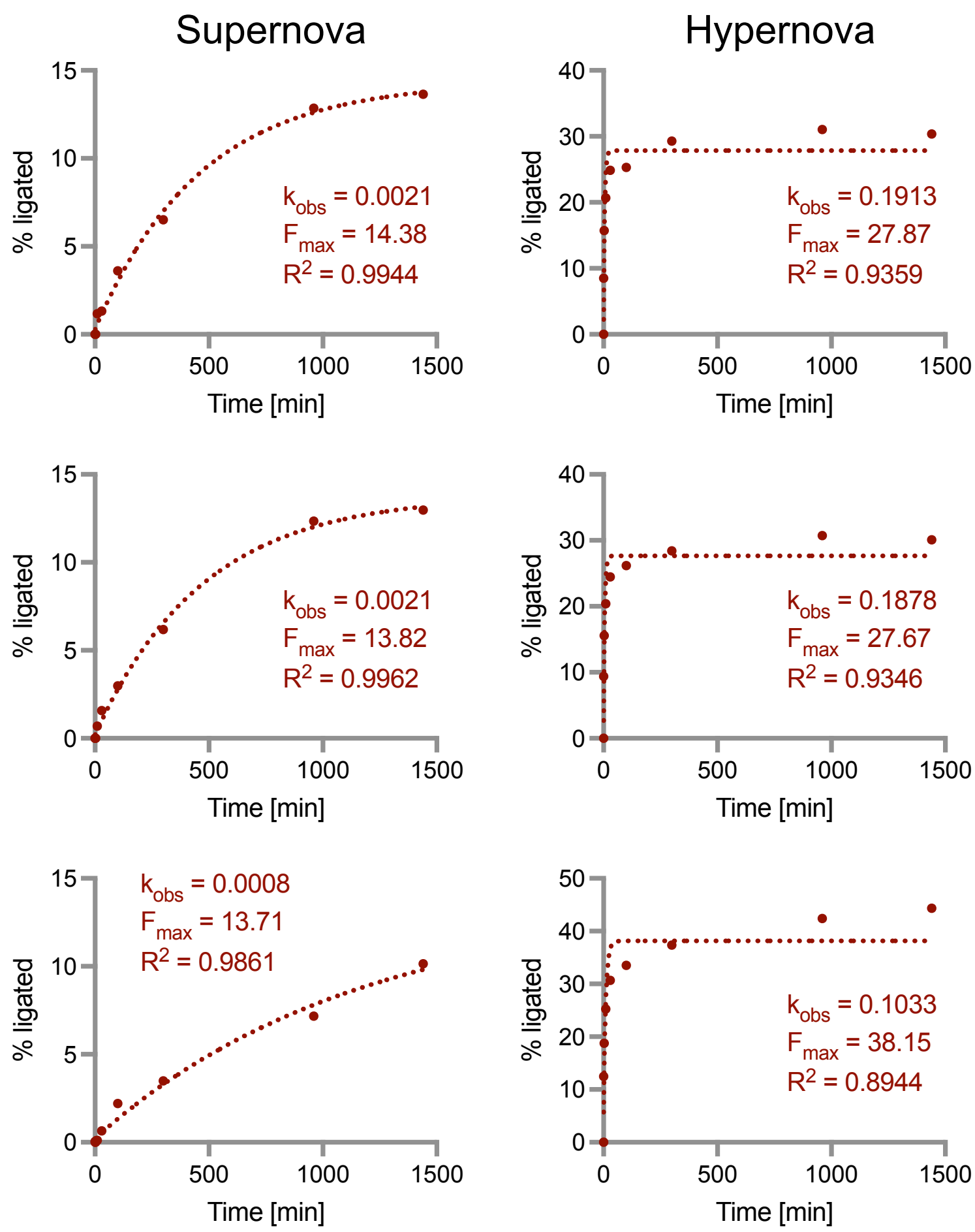

Supplementary Figure S15. Head-to-head comparison of the activity of Supernova (left) and Hypernova (right) over 24 hours. Conditions were 1 µM deoxyribozyme and 1 µM CDP-Star in 1× selection buffer. After incubating for the indicated time, activity was measured using the ligation assay, and fitted using the equation $P = F \times (1 - e^{-kt})$, where P is the percent ligated, F_{max} is the maximum percent ligated, k_{obs} is the rate constant, and t is time. k_{obs} of Supernova = 0.0017 ± 0.0008 min^{-1}; F_{max} of Supernova = 14 ± 0.4; k_{obs} of Hypernova = 0.16 ± 0.05 min^{-1}; F_{max} of Hypernova = 31 ± 6.

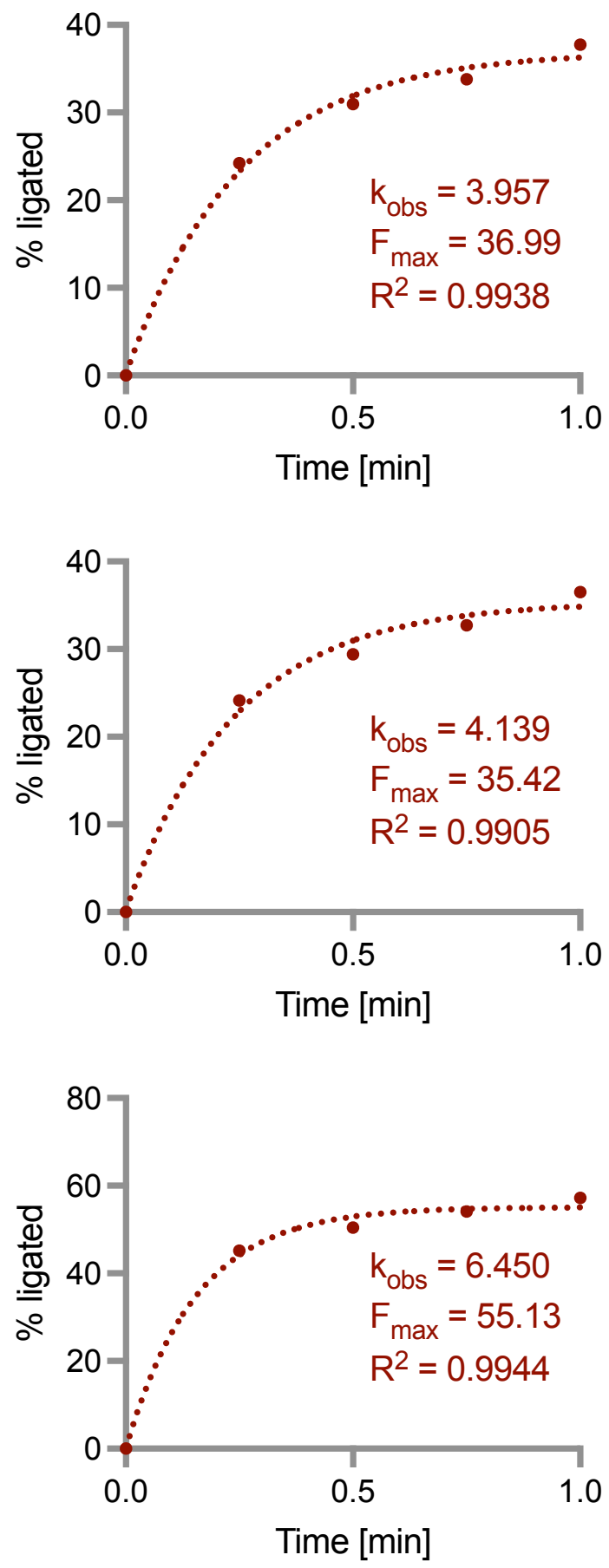

Supplementary Figure S16. Estimating the lower limit of the k_{cat} of Hypernova. Reactions were performed as described in the Materials and Methods. Conditions were 1 µM deoxyribozyme and 100 µM CDP-Star in 1× selection buffer. After incubating for the indicated time, activity was measured using the ligation assay, and fitted using the equation $P = F \times (1 - e^{-kt})$, where P is the percent ligated, F_{max} is the maximum percent ligated, k_{obs} is the rate constant, and t is time.

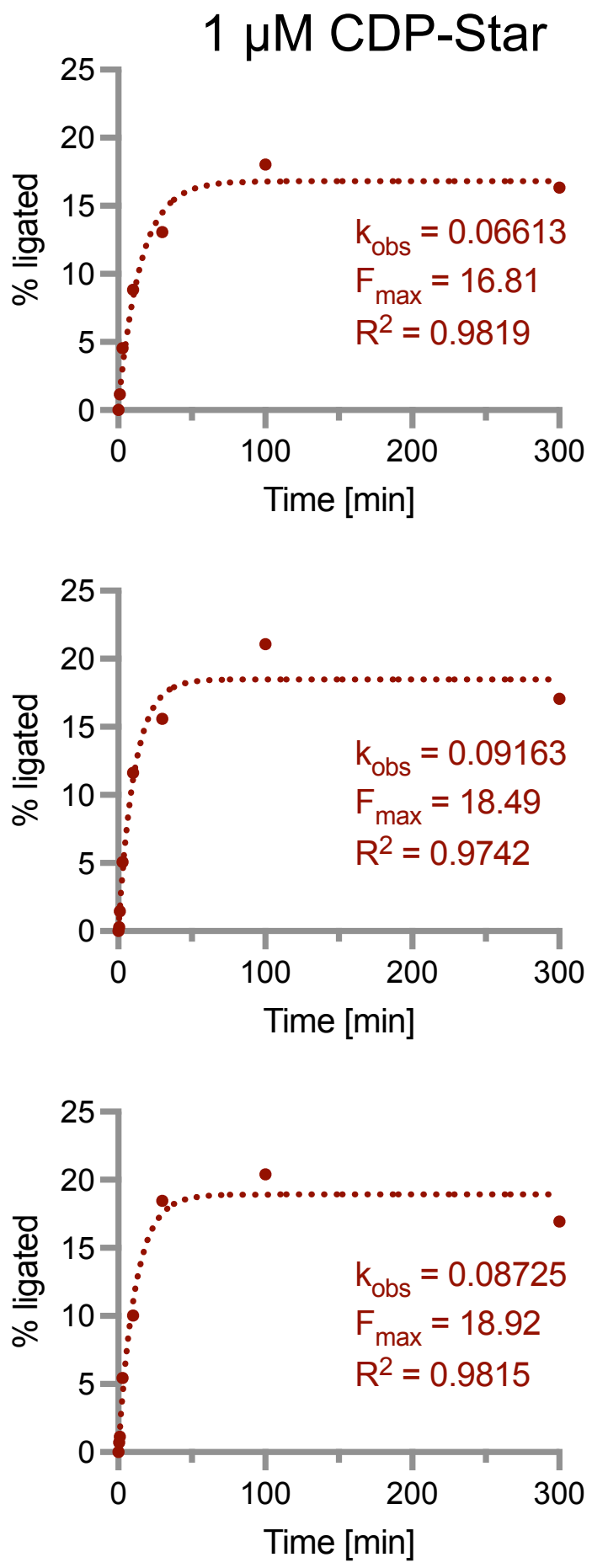

Supplementary Figure S17. Kinetic characterization of fluorophore-labeled Hypernova at 1 μM CDP-Star. Reactions were performed as described in the Materials and Methods. Conditions were 40 nM fluorophore-labeled Hypernova and 1 μM CDP-Star in 1× selection buffer. After incubating for the indicated time, activity was measured using the ligation assay, and fitted using the equation $P = F \times (1 - e^{-kt})$, where P is the percent ligated, F_{max} is the maximum percent ligated, k_{obs} is the rate constant, and t is time. The 1 μM point in Figure 6d (0.08 ± 0.01 min^{-1}) is the average of these three measurements.

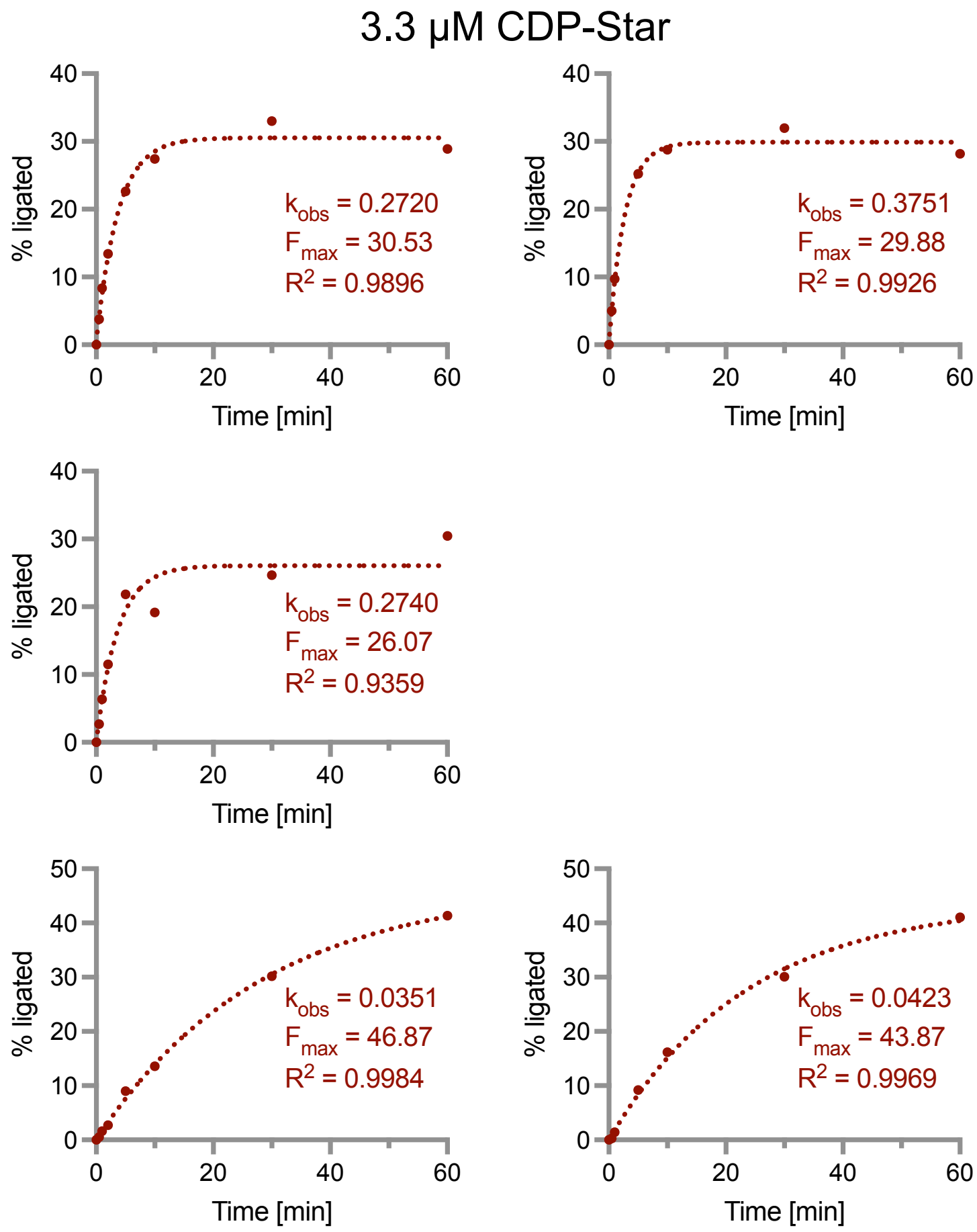

Supplementary Figure S18. Kinetic characterization of fluorophore-labeled Hypernova at 3.3 µM CDP-Star. Reactions were performed as described in the Materials and Methods. Conditions were 40 nM fluorophore-labeled Hypernova and 3.3 µM CDP-Star in 1× selection buffer. After incubating for the indicated time, activity was measured using the ligation assay, and fitted using the equation $P = F \times (1 - e^{-kt})$, where P is the percent ligated, F_{max} is the maximum percent ligated, k_{obs} is the rate constant, and t is time. The 3.3 µM point in Figure 6d (0.2 ± 0.15 min^{-1}) is the average of these five measurements.

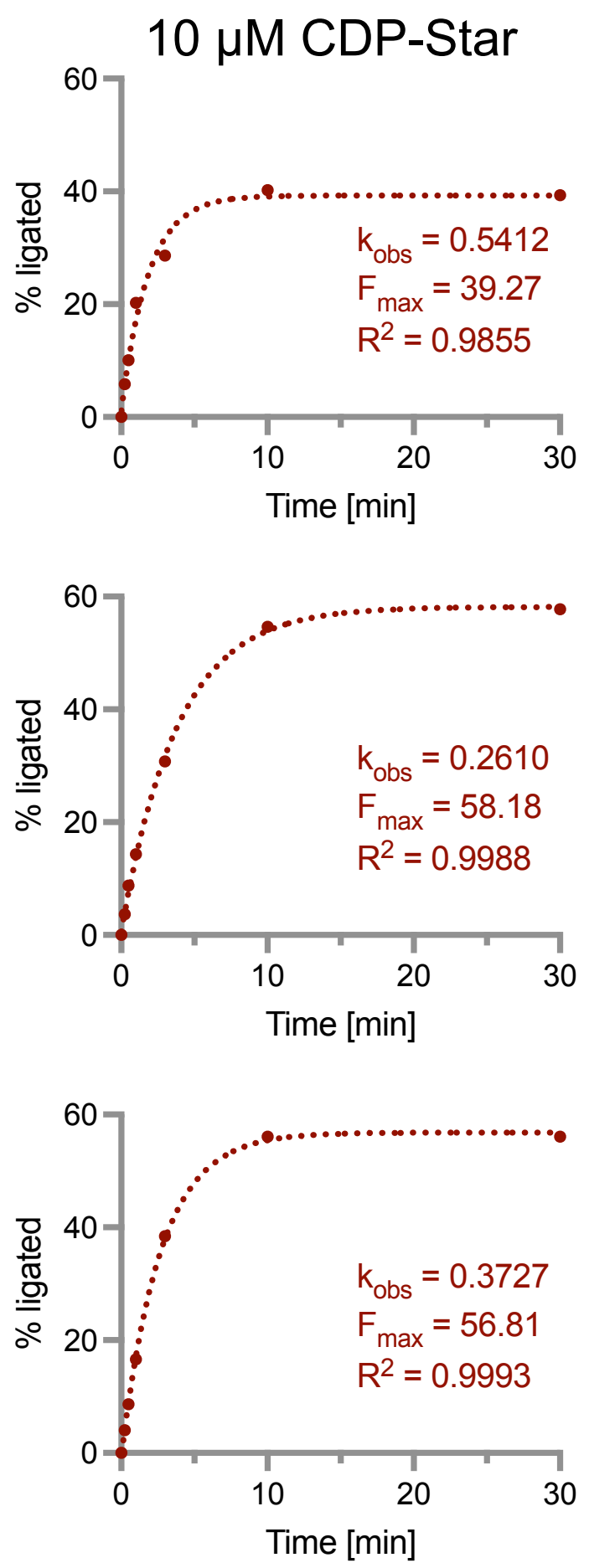

Supplementary Figure S19. Kinetic characterization of fluorophore-labeled Hypernova at 10 μM CDP-Star. Reactions were performed as described in the Materials and Methods. Conditions were 40 nM fluorophore-labeled Hypernova and 10 μM CDP-Star in 1× selection buffer. After incubating for the indicated time, activity was measured using the ligation assay, and fitted using the equation $P = F \times (1 - e^{-kt})$, where P is the percent ligated, F_{max} is the maximum percent ligated, k_{obs} is the rate constant, and t is time. The 10 μM point in Figure 6d (0.39 ± 0.14 min^{-1}) is the average of these three measurements.

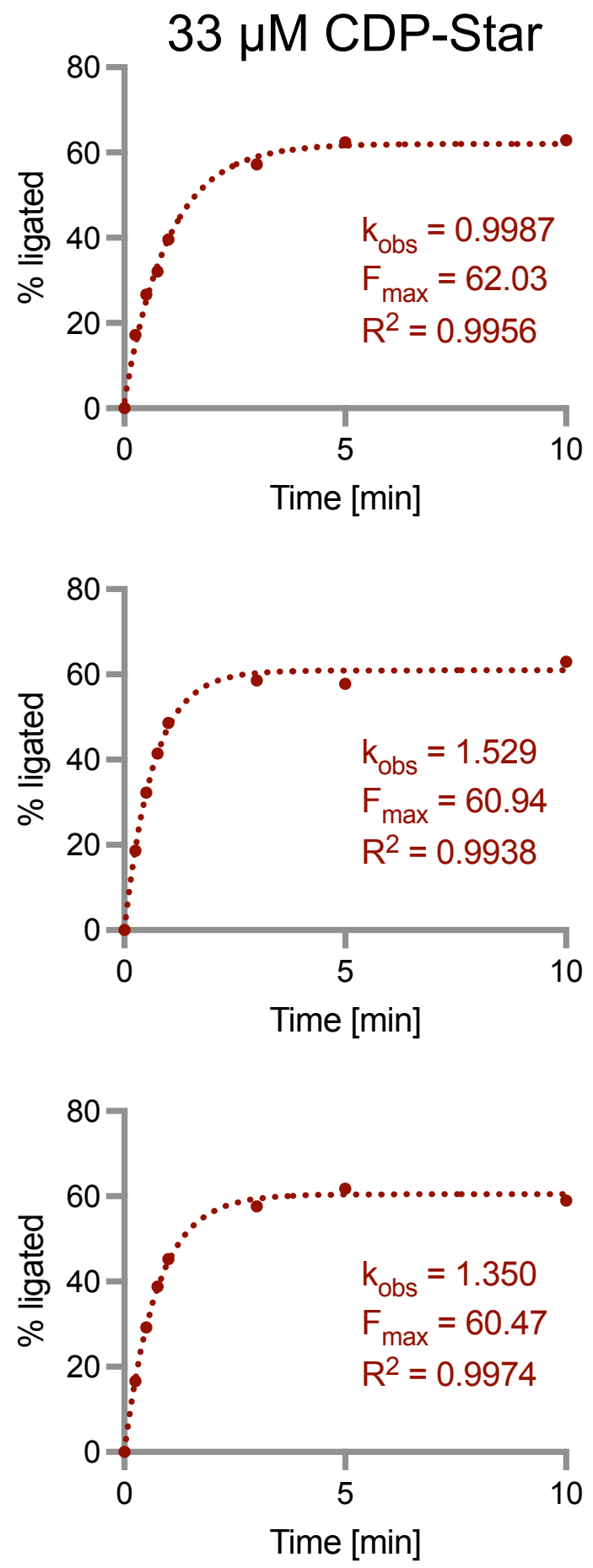

Supplementary Figure S20. Kinetic characterization of fluorophore-labeled Hypernova at 33 µM CDP-Star. Reactions were performed as described in the Materials and Methods. Conditions were 40 nM fluorophore-labeled Hypernova and 33 µM CDP-Star in 1× selection buffer. After incubating for the indicated time, activity was measured using the ligation assay, and fitted using the equation $P = F \times (1 - e^{-kt})$, where P is the percent ligated, F_{max} is the maximum percent ligated, k_{obs} is the rate constant, and t is time. The 33 µM point in Figure 6d (1.3 ± 0.3 min^{-1}) is the average of these three measurements.

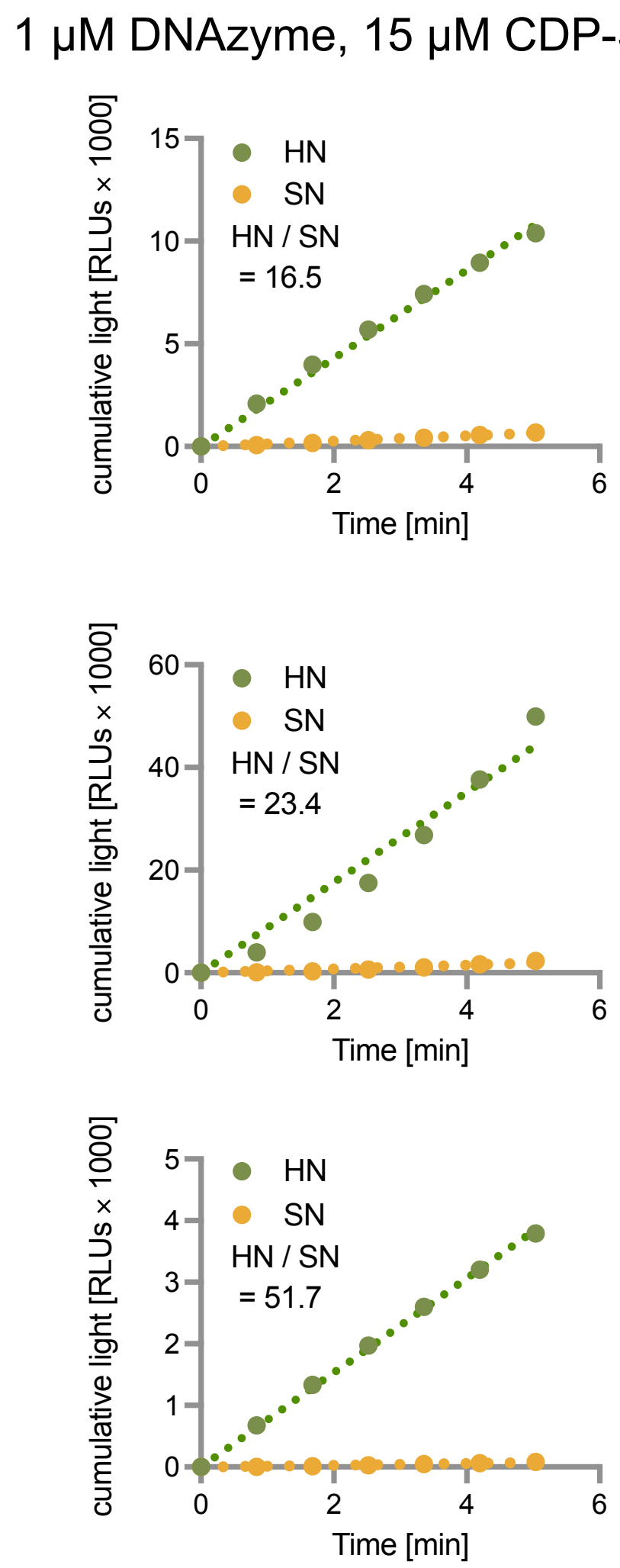

Supplementary Figure S21. Comparison of the initial rate of light production of Supernova and Hypernova at 1 µM deoxyribozyme and 15 µM CDP-Star. Reactions were performed as described in the Materials and Methods. Conditions were 1 µM Supernova or Hypernova and 15 µM CDP-Star in 1× selection buffer. HN / SN = the initial rate of light production of Hypernova divided by the initial rate of light production of Supernova. Note that each graph shows a comparison of time courses performed at the same time, which helps to reduce variation in these experiments. The initial rate of light production of Hypernova is 30 ± 20 fold faster than Supernova under these conditions.

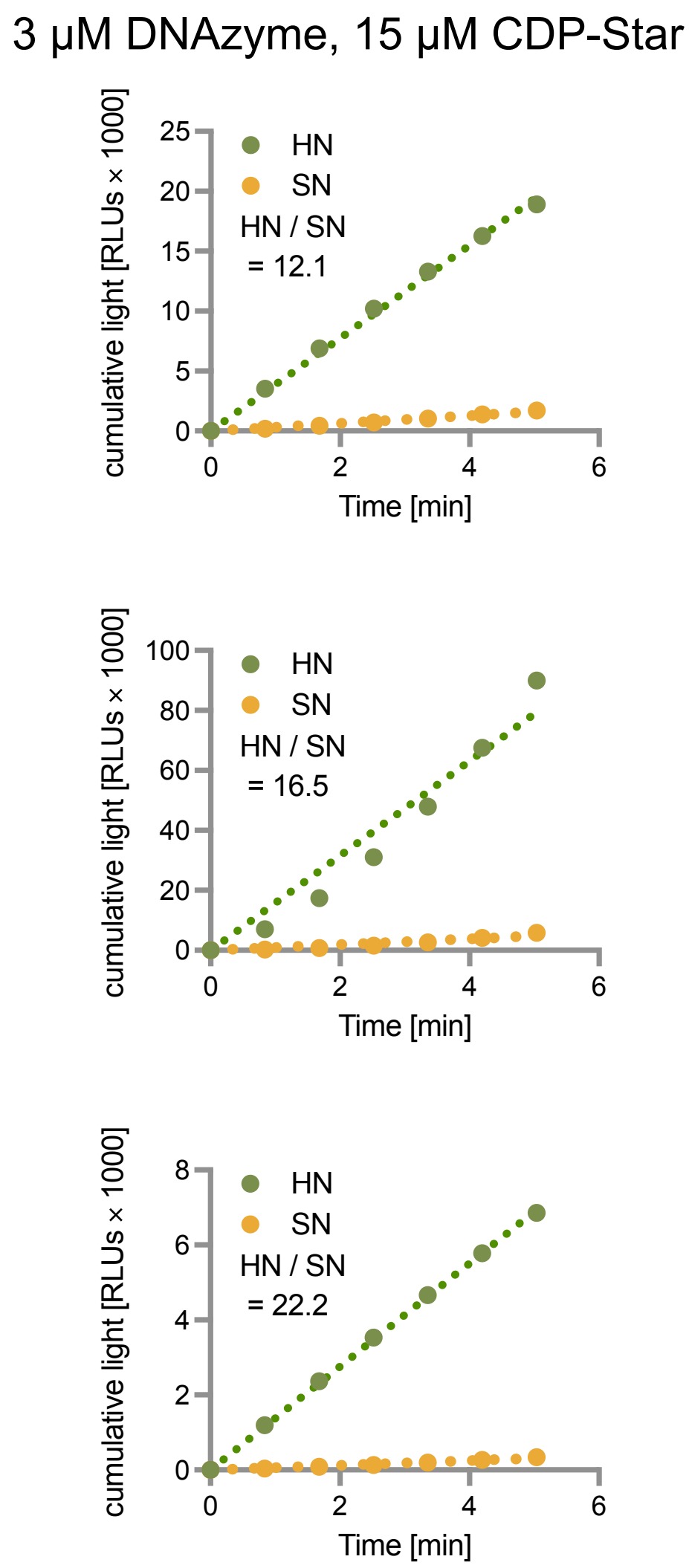

Supplementary Figure S22. Comparison of the initial rate of light production of Supernova and Hypernova at 3 µM deoxyribozyme and 15 µM CDP-Star. Reactions were performed as described in the Materials and Methods. Conditions were 3 µM Supernova or Hypernova and 15 µM CDP-Star in 1× selection buffer. HN / SN = the initial rate of light production of Hypernova divided by the initial rate of light production of Supernova. Note that each graph shows a comparison of time courses performed at the same time, which helps to reduce variation in these experiments. The initial rate of light production of Hypernova is 17 ± 5 fold faster than Supernova under these conditions.

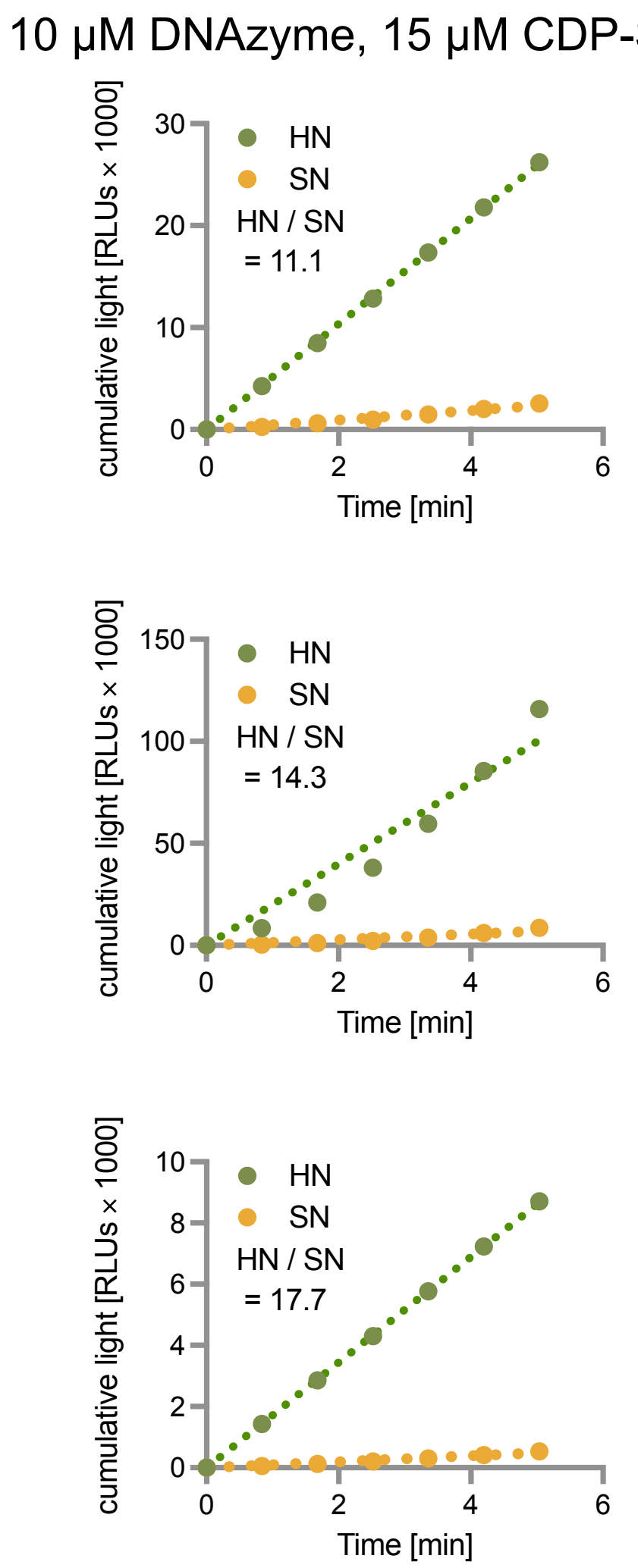

Supplementary Figure S23. Comparison of the initial rate of light production of Supernova and Hypernova at 10 µM deoxyribozyme and 15 µM CDP-Star. Reactions were performed as described in the Materials and Methods. Conditions were 10 µM Supernova or Hypernova and 15 µM CDP-Star in 1× selection buffer. HN / SN = the initial rate of light production of Hypernova divided by the initial rate of light production of Supernova. Note that each graph shows a comparison of time courses performed at the same time, which helps to reduce variation in these experiments. The initial rate of light production of Hypernova is 14 ± 3 fold faster than Supernova under these conditions.

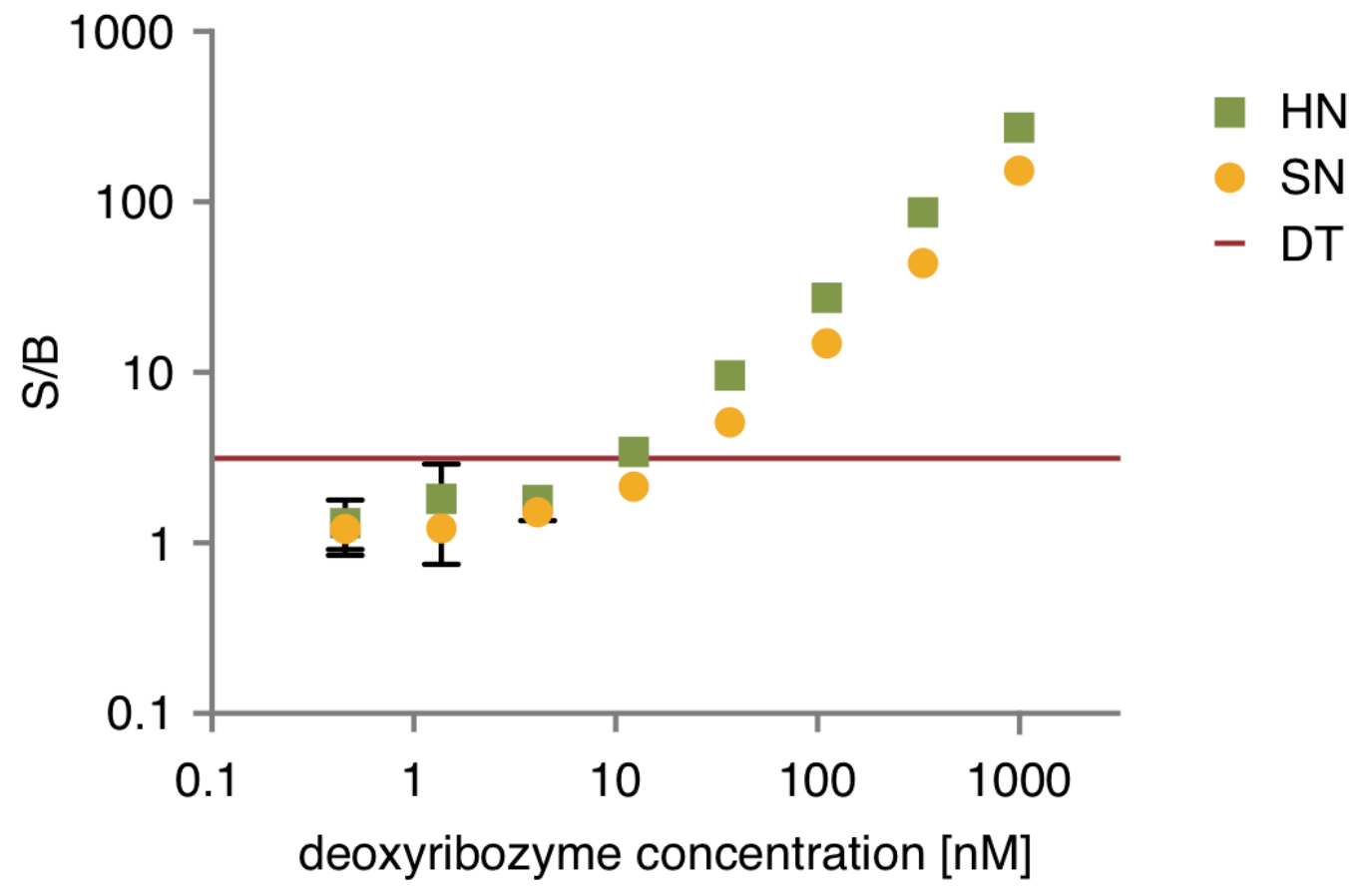

Supplementary Figure S24. Limit of Detection of Hypernova. The limit of detection is the concentration of deoxyribozyme at which there is at least a 95% probability that the signal is higher than that of the background (CDP-Star in selection buffer). This was calculated as DT = NC + 3×StDev where NC is the average signal of background. Error bars show standard deviation of three measurements. HN = Hypernova; SN = Supernova; DT = detection threshold.

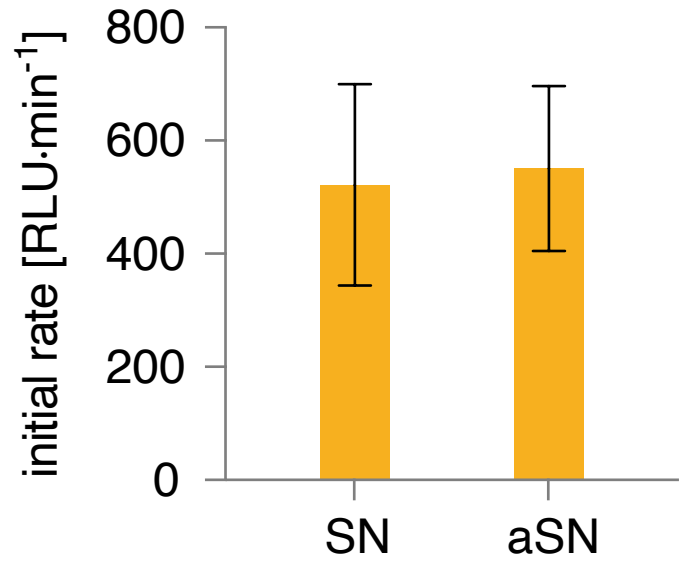

Supplementary Figure S25. Initial rate of light production of Supernova (SN) and an engineered version of Supernova called aSN. Both aSN and Hypernova contain two TC motifs (the preferred editing site of APOBEC3A), whereas Supernova contains only one.

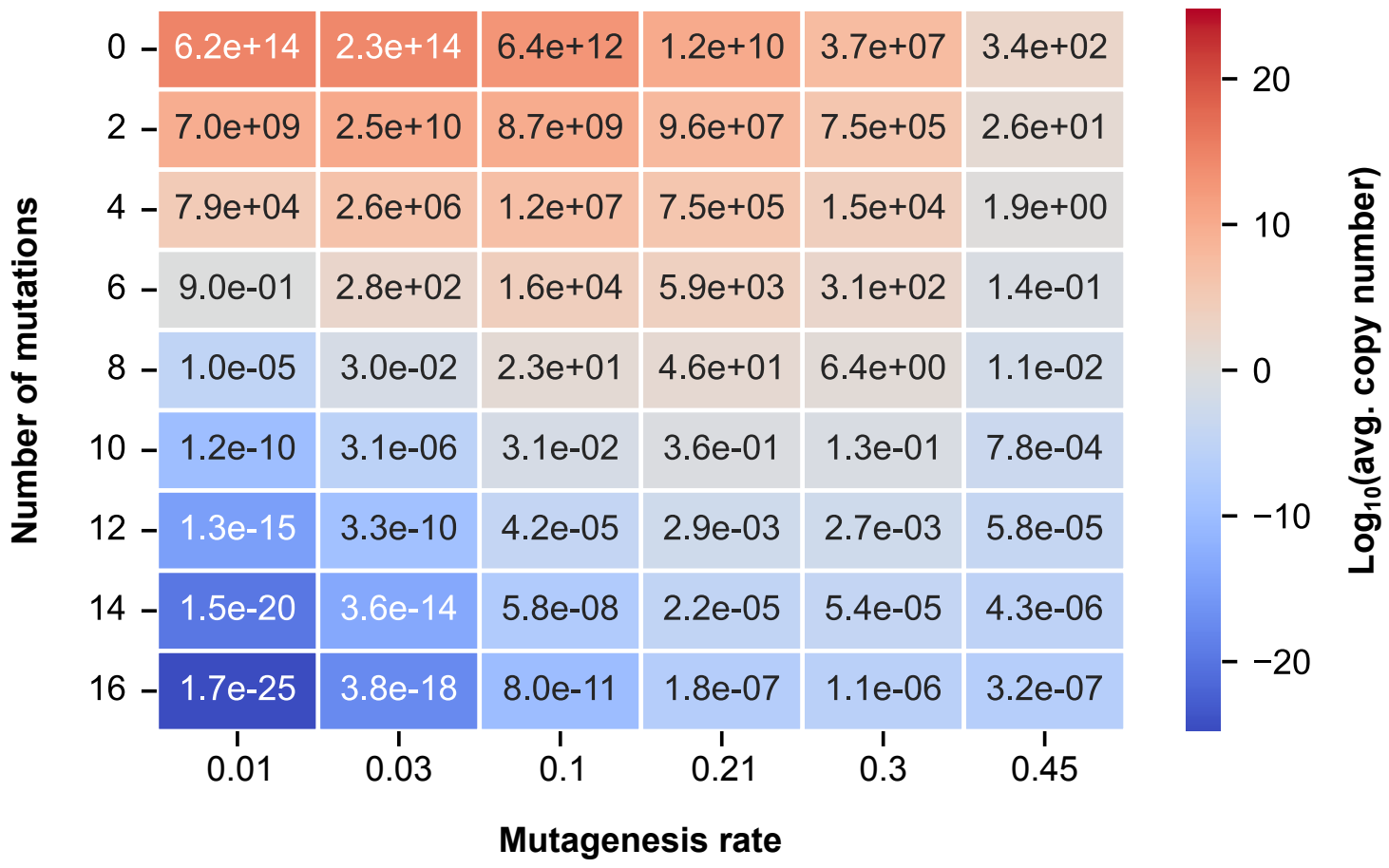

Supplementary Figure S26: Calculated average sequence copy number as a function of Hamming distance and mutagenesis rate. Heatmap showing the calculated average copy number per unique sequence at Hamming distance *d* from the parent sequence under mutagenesis rate *m*. Average copy number is modeled as $C(d,m) = \frac{N \cdot P(d,m)}{S(d)}$ where $P(d,m) = \binom{L}{d} m^d (1-m)^{L-d}$ is the probability of a sequence occurring at distance *d*, $S(d) = \binom{L}{d} 3^d$ is the size of the sequence space at distance *d*, *L* is sequence length, and *N* is the total library size.

Supplementary Table S1. Oligonucleotides used in this study.

Supernova	GGAAGAAAAAGAATATCCCCAAAAGGGGAGTGACTTGGGATGGGGG
Hypernova	GGCAGACACAGAATATCTCTTACAAGAGAGTGACTTGGGATGAGTA
Cy3-labeled Hypernova	GGCAGACACAGAATATCTCTTACAAGAGAGTGACTTGGGATGAGTAAAA-Cy3
Splint for SN	TATTCTTTTTCTTCCTGATACTACACCTGAGCGGT
Secondary structure library	GGMAGWAAAAKNDNDWYYYYAAAAGGGGWRTGACYTGGGATGGGGNAACGCTTGCTCCGGTCTGATGT
	GGMAGWAAAAKNDNDWTYYYAAAAGGGAWRTGACYTGGGATWGGGNAACGCTTGCTCCGGTCTGATGT
	GGMAGWAAAAKNDNDWYTYYAAAAGGAGWRTGACYTGGGATGWGGNAACGCTTGCTCCGGTCTGATGT
	GGMAGWAAAAKNDNDWYYTYAAAAGAGGWRTGACYTGGGATGGWGNAACGCTTGCTCCGGTCTGATGT
	GGMAGWAAAAKNDNDWYYYTAAAAAGGGWRTGACYTGGGATGGGWNAACGCTTGCTCCGGTCTGATGT
	GGMAGWAAAAKNDNDWTTYYAAAAGGAAWRTGACYTGGGATWWGGNAACGCTTGCTCCGGTCTGATGT
	GGMAGWAAAAKNDNDWTYTYAAAAGAGAWRTGACYTGGGATWGWGNAACGCTTGCTCCGGTCTGATGT
	GGMAGWAAAAKNDNDWTYYTAAAAAGGAWRTGACYTGGGATWGGWNAACGCTTGCTCCGGTCTGATGT
	GGMAGWAAAAKNDNDWYTTYAAAAGAAGWRTGACYTGGGATGWWGNAACGCTTGCTCCGGTCTGATGT
	GGMAGWAAAAKNDNDWYTYTAAAAAGAGWRTGACYTGGGATGWGWNAACGCTTGCTCCGGTCTGATGT
	GGMAGWAAAAKNDNDWYYTTAAAAAAGGWRTGACYTGGGATGGWWNAACGCTTGCTCCGGTCTGATGT
	GGMAGWAAAAKNDNDWTTTYAAAAGAAAWRTGACYTGGGATWWWGNAACGCTTGCTCCGGTCTGATGT
	GGMAGWAAAAKNDNDWTTYTAAAAAGAAWRTGACYTGGGATWWGWNAACGCTTGCTCCGGTCTGATGT
	GGMAGWAAAAKNDNDWTYTTAAAAAAGAWRTGACYTGGGATWGWWNAACGCTTGCTCCGGTCTGATGT
	GGMAGWAAAAKNDNDWYTTTAAAAAAAGWRTGACYTGGGATGWWWNAACGCTTGCTCCGGTCTGATGT
	GGMAGWAAAAKNDNDWTTTTAAAAAAAAWRTGACYTGGGATWWWWNAACGCTTGCTCCGGTCTGATGT
Splint1	HNHNMTTTTWCTKCCCGCAGGGACTTCAGTGGATC
Fwd1	GATCCACTGAAGTCCCTGCG
Rev1	ACATCAGACCGGAGCAAGCG
**Secondary structure selection fitness landscape analysis (cpm)**	
1 Rnd 1	GGCAGAAAAAGAATTTCCCTAAAAGGGAGTGACTTGGGATGGGTG
2 Rnd 1	GGCAGAAAAAGAATTTCCCTAAAAAGGGAGTGACTTGGGATGGGTT
3 Rnd 1	GGCAGAAAAAGAATATTCCTAAAAAGGGAGTGACTTGGGATGGGTG
4 Rnd 1	GGCAGAAAAAGAATATCCTTAAAAAAGGAGTGACTTGGGATGGTTG
5 Rnd 1	GGCAGTAAAAGAATTTCTCTAAAAGGAGAGTGACTTGGGATGAGGT
6 Rnd 1	GGCAGTAAAAGAATATTCCTAAAAAGGGAGTGACTTGGGATGGGTG
7 Rnd 1	GGCAGTAAAAGAATTTCTCCAAAAGGAGAGTGACTTGGGATGAGGG
8 Rnd 1	GGCAGTAAAATTGAATTTCCAAAAGGGGAGTGACTTGGGATGGGGT
9 Rnd 1	GGCAGTAAAAGAATATCTCCAAAAGGAGAGTGACTTGGGATGAGGG
10 Rnd 1	GGCAGTAAAAGAATTTTCCTAAAAAGGGAGTGACTTGGGATGGGTG
11 Rnd 1	GGCAGTAAAAGAATATCTTTAAAAAGAGAGTGACTTGGGATGAGTT
12 Rnd 1	GGCAGTAAAAGAATTTCCTTAAAAAGGGAGTGACTTGGGATGGGTT
13 Rnd 1	GGCAGTAAAATGAATTTTCCAAAAGGGGAGTGACTTGGGATGGGGT

14 Rnd 1	GGCAGTAAAAGAATTTCCTTAAAAGAGGAGTGACTTGGGATGGTGT
15 Rnd 1	GGCAGTAAAATGAATTTCCCAAAAGGGGAGTGACTTGGGATGGGGT
16 Rnd 1	GGCAGTAAAAGAATATCTCTAAAAGGAGAGTGACTTGGGATGTGGT
17 Rnd 1	GGCAGTAAAAGAATATTTCCAAAAGGAGAGTGACTTGGGATGAGGT
18 Rnd 1	GGCAGTAAAAGAATATCTTCAAAAGGAGAGTGACTTGGGATGTGGG
19 Rnd 1	GGCAGTAAAAGAATTATCCTAAAAAGGGAGTGACTTGGGATGGGTT
20 Rnd 1	GGCAGTAAAAGAATTTCTCTAAAAAGGGAGTGACTTGGGATGGGTG
21 Rnd 1	GGCAGTAAAAGAATTTTCCTAAAAAGGGAGTGACTTGGGATGGGAT
22 Rnd 1	GGCAGTAAAAGAATTACTCCAAAAGGAGAGTGACTTGGGATGAGGT
23 Rnd 1	GGCAGTAAAATGAATTTCCTAAAAAGGGAGTGACTTGGGATGGGTT
24 Rnd 1	GGCAGTAAAATGGAATTTCCAAAAGGGGAGTGACTTGGGATGGGGT
25 Rnd 1	GGCAGTAAAAGGAATATTCCAAAAGGGGAGTGACTTGGGATGGGGA
26 Rnd 1	GGCAGTAAAATTGAATTTCCAAAAGGGGAGTGACTTGGGATGGGGC
27 Rnd 1	GGCAGTAAAATTTTTTTTTTAAAAGGGGTATGACTTGGGATGGGGT
28 Rnd 1	GGCAGTAAAATTTTTTTTTTAAAAGGAATGTGACTTGGGATTTGGC
29 Rnd 1	TGCAGTAAAATATAAATTCTAAAAGGAAAGTGACTTGGGATATGGT
30 Rnd 1	TGCAGTAAAATTTTTTTTTTAAAAAAGATGTGACTTGGGATTGAAG
1 Rnd 2	GGCAGAAAAAGAATTTCCCTAAAAAGGGAGTGACTTGGGATGGGTT
2 Rnd 2	GGCAGAAAAAGAATATCCTTAAAAAAGGAGTGACTTGGGATGGATT
3 Rnd 2	GGAAGAAAAAGAATATCTCTAAAAAGAGAGTGACTTGGGATGAGTT
4 Rnd 2	GGCAGTAAAAGAATATCTCTAAAAGGAGAGTGACTTGGGATGAGGC
5 Rnd 2	GGCAGAAAAAGAATATCCTTAAAAAAGGAGTGACTTGGGATGGATA
6 Rnd 2	GGCAGAAAAAGAATATCTCTAAAAAGAGAGTGACTTGGGATGAGAA
7 Rnd 2	GGCAGTAAAAGAATATCCTTAAAAGAGGAGTGACTTGGGATGGAGT
8 Rnd 2	GGCAGTAAAAGAATTACTCCAAAAGGAGAGTGACTTGGGATGAGGT
9 Rnd 2	GGAAGTAAAAGAATATCTCTAAAAGGAGAGTGACTTGGGATGAGGG
10 Rnd 2	GGAAGAAAAAGGGATATCCCAAAAGGGGAGTGACTTGGGATGGGGG
11 Rnd 2	GGCAGAAAAAGAATATCCTTAAAAGAGGAGTGACTTGGGATGGAGG
12 Rnd 2	GGAAGAAAAAGAATATCCTTAAAAGAGGAGTGACTTGGGATGGTGA
13 Rnd 2	GGAAGAAAAAGAATATCTCTAAAAAGGGAGTGACTTGGGATGGGTG
14 Rnd 2	GGCAGTAAAAGAATATTCCCAAAAGGGAAGTGACTTGGGATAGGGT
15 Rnd 2	GGCAGTAAAAGAATATCCTCAAAAGAGGAGTGACTTGGGATGGTGA
16 Rnd 2	GGCAGTAAAAGAATATTCTCAAAAGAGAAGTGACTTGGGATTGAGT
17 Rnd 2	GGCAGTAAAAGAATATTCCCAAAAGGGGAGTGACTTGGGATGGGGC
18 Rnd 2	GGCAGAAAAATGAATTTTCCAAAAGGGAAGTGACTTGGGATTGGGT
19 Rnd 2	GGAAGTAAAAGAATTTCCCCAAAAGGGGAGTGACTTGGGATGGGGG
20 Rnd 2	GGCAGTAAAAGAATTTTCCTAAAAAGGGAGTGACTTGGGATGGGTA
21 Rnd 2	GGCAGAAAAAGAACTTCTCTAAAAAGAGAGTGACTTGGGATGAGTG
22 Rnd 2	GGCAGTAAAAGAATTTTCCCAAAAGGGGAGTGACTTGGGATGGGGT
23 Rnd 2	GGCAGAAAAAGGGATTTCCTAAAAAGGGAGTGACTTGGGATGGGTG
24 Rnd 2	GGCAGTAAAAGAATTACCCTAAAAAGGGAGTGACTTGGGATGGGAT
25 Rnd 2	GGCAGTAAAAGAATGTCCCTAAAAGGGGAATGACTTGGGATGGGGT
26 Rnd 2	GGCAGTAAAAGAACATCTCTAAAAAGGGAGTGACTTGGGATGGGTT
27 Rnd 2	GGCAGAAAAATGGATATTCCAAAAGGGGAGTGACTTGGGATGGGGC
28 Rnd 2	GGCAGTAAAATGAATATTCCAAAAGGGGAGTGACTTGGGATGGGGC
29 Rnd 2	GGCAGTAAAATTGAATTTCCAAAAGGGAAGTGACTTGGGATAGGGT
30 Rnd 2	TGCAGTAAAATTTTTATTTCAAAAGGGGTATGACCTGGGATGGGGC
1 Rnd 3	GGCAGAAAAAGAATATCTCTAAAAAGAGAGTGACTTGGGATGAGTG
2 Rnd 3	GGCAGAAAAATGAATTTCCCAAAAGGGGAGTGACTTGGGATGGGGT
3 Rnd 3	GGAAGAAAAAGAATATCCCCAAAAGGGGAGTGACTTGGGATGGGGT
4 Rnd 3	GGCAGTAAAAGAATATCCCTAAAAAGGGAGTGACTTGGGATGGGTC
5 Rnd 3	GGAAGTAAAAGAATATCTCCAAAAGGAGAGTGACTTGGGATGAGGG
6 Rnd 3	GGCAGTAAAAGAATATCCCCAAAAGGGGAGTGACTTGGGATGGGGA
7 Rnd 3	GGAAGTAAAAGAATATCTCCAAAAGGAGAGTGACTTGGGATGAGGC
8 Rnd 3	GGCAGTAAAAGAATATCTTTAAAAAGAGAGTGACTTGGGATGAGTT
9 Rnd 3	GGAAGTAAAAGAATTTCTCCAAAAGGAGAGTGACTTGGGATGAGGA

10 Rnd 3	GGCAGTAAAAGAATATCTTTAAAAAAAGAGTGACTTGGGATGATTA
11 Rnd 3	GGCAGTAAAAGAACATCTCTAAAAAGAGAGTGACTTGGGATGAGTT
12 Rnd 3	GGCAGTAAAAGAATGTCTCCAAAAGGAGAATGACTTGGGATGAGGT
13 Rnd 3	GGCAGAAAAAGAACATCTCCAAAAGGAGAGTGACTTGGGATGAGGC
14 Rnd 3	GGCAGAAAAAGGGATATCCCAAAAGGGGAGTGACTTGGGATGGGGA
15 Rnd 3	GGCAGAAAAAGAATATCCTTAAAAAGGGAGTGACTTGGGATGGGTG
16 Rnd 3	GGCAGAAAAAGAATATTCTCAAAAGAGAAGTGACTTGGGATAGAGT
17 Rnd 3	GGCAGAAAAAGAATTTTCCTAAAAGGGGAGTGACTTGGGATGGGGT
18 Rnd 3	GGAAGAAAAAGAATTTCCCCAAAAGGGGAGTGACTTGGGATGGGGC
19 Rnd 3	GGAAGAAAAAGAATATCCTTAAAAAAAGGAGTGACTTGGGATGGAAT
20 Rnd 3	GGAAGTAAAAGAATTTCCTTAAAAAGGGAGTGACTTGGGATGGGTT
21 Rnd 3	GGCAGTAAAAGAATAACTCCAAAAGGAGAGTGACTTGGGATGAGGT
22 Rnd 3	GGAAGTAAAAGAATATCTTCAAAAGAAGAGTGACTTGGGATGAAGT
23 Rnd 3	GGAAGAAAAATGAATATTCCAAAAGGGGAGTGACTTGGGATGGGGG
24 Rnd 3	GGCAGTAAAAGAATTTCCTTAAAAGAGGAGTGACTTGGGATGGAGT
25 Rnd 3	GGCAGTAAAAGAATAACTCTAAAAAGAGAGTGACTTGGGATGAGTT
26 Rnd 3	GGCAGAAAAAGAATATCCTTAAAAGGGGAGTGACTTGGGATGGGGC
27 Rnd 3	GGAAGTAAAAGAAGATCTCTAAAAAGAGAGTGACTTGGGATGAGTG
28 Rnd 3	GGCAGTAAAAGAATTTCTCTAAAAAGAGAGTGACTTGGGATGAGGT
29 Rnd 3	GGCAGTAAAATGAATTCTCCAAAAGGGGAGTGACTTGGGATGGGGT
30 Rnd 3	TGCAGTAAAATTTTAACTTTAAAAAAAGAATGACCTGGGATGTTAG
1 Rnd 4	GGCAGAAAAAGAATATCTCTAAAAAGAGAGTGACTTGGGATGAGTG
2 Rnd 4	GGCAGTAAAAGAATTTCCCTAAAAAGGGAGTGACTTGGGATGGGTT
3 Rnd 4	GGCAGAAAAAGAATATTCCTAAAAAGGGAGTGACTTGGGATGGGTG
4 Rnd 4	GGAAGAAAAAGAATATCTCCAAAAGGAGAGTGACTTGGGATGAGGC
5 Rnd 4	GGCAGAAAAAGAATATCTTCAAAAGAAGAGTGACTTGGGATGATGG
6 Rnd 4	GGCAGAAAAATAGAATTTCCAAAAGGGGAGTGACTTGGGATGGGGT
7 Rnd 4	GGAAGAAAAAGAATATTCCTAAAAAGGGAGTGACTTGGGATGGGTG
8 Rnd 4	GGCAGAAAAAGAATATTCCTAAAAAGGGAGTGACTTGGGATGGGAT
9 Rnd 4	GGCAGTAAAAGAATATCTTTAAAAGAAGAGTGACTTGGGATGATGT
10 Rnd 4	GGAAGAAAAAGAATATCCTTAAAAGAGGAGTGACTTGGGATGGTGT
11 Rnd 4	GGAAGTAAAAGAATATCCCTAAAAAGGGAGTGACTTGGGATGGGTT
12 Rnd 4	GGAAGAAAAAGAATATCTCTAAAAAGAGAGTGACTTGGGATGAGAG
13 Rnd 4	GGCAGAAAAAGGAATTTCCCAAAAGGGGAGTGACTTGGGATGGGGT
14 Rnd 4	GGCAGAAAAAGAATATCCTTAAAAGAGGAGTGACTTGGGATGGTGA
15 Rnd 4	GGCAGTAAAAGAATTTCCTTAAAAAGGGAGTGACTTGGGATGGGTT
16 Rnd 4	GGCAGAAAAAGAATATTCTTAAAAAAGAAGTGACTTGGGATTGATT
17 Rnd 4	GGCAGAAAAAGAATATCTCTAAAAAGAGAGTGACTTGGGATGGGTT
18 Rnd 4	GGCAGAAAAAGAATATCTCCAAAAGGAGAGTGACCTGGGATGAGGG
19 Rnd 4	GGAAGAAAAATGAATTTCCCAAAAGGGGAGTGACTTGGGATGGGGC
20 Rnd 4	GGCAGTAAAAGAACTTCTCTAAAAAGAGAGTGACTTGGGATGAGTT
21 Rnd 4	GGCAGAAAAAGAATATCTCCAAAAGGAGAGTGACTTGGGATGAGGT
22 Rnd 4	GGAAGTAAAAGAACATCTCTAAAAAGAGAGTGACTTGGGATGAGTG
23 Rnd 4	GGAAGTAAAAGAATATCTTTAAAAGAAGAGTGACTTGGGATGATGT
24 Rnd 4	GGAAGAAAAAGAATATTCCCAAAAGGGGAGTGACTTGGGATGGGGC
25 Rnd 4	GGCAGTAAAAGAATATCTTTAAAAGAAGAGTGACTTGGGATGATGC
26 Rnd 4	GGAAGAAAAAGTGAATTTCCAAAAGGGGAGTGACTTGGGATGGGGT
27 Rnd 4	GGCAGAAAAATGAATTTCCCAAAAGGGGAGTGACTTGGGATGGGTT
28 Rnd 4	GGCAGTAAAAGAATTTCCCTAAAAAGGGAGTGACTTGGGATGAGTG
29 Rnd 4	GGCAGTAAAAGAATTTCTCTAAAAAGAGAGTGACCTGGGATGAGTT
30 Rnd 4	TGCAGTAAAATTTATTTCTTAAAAAAGGTATGACTTGGGATGGATG
**Secondary structure selection fitness landscape analysis (enrichment)**	
1 Rnd 1	GGCAGTAAAAGAATTTCCCTAAAAAGGGAGTGACTTGGGATGGGTT
2 Rnd 1	GGCAGTAAAAGAATTTCCCCAAAAGGGGAGTGACTTGGGATGGGGT
3 Rnd 1	GGCAGTAAAAGAATTTCCCTAAAAAGGGAGTGACTTGGGATGGGTG
4 Rnd 1	GGCAGTAAAAGAATTTCCCTAAAAAGGGAGTGACTTGGGATGGGTA

5 Rnd 1	GGCAGAAAAAGAATTTCCCTAAAAAGGGAGTGACTTGGGATGGGTT
6 Rnd 1	GGCAGAAAAAGAATTTCCCCAAAAGGGGAGTGACTTGGGATGGGGT
7 Rnd 1	GGCAGAAAAAGAATTTCCCTAAAAAGGGAGTGACTTGGGATGGGTG
8 Rnd 1	GGCAGTAAAAGAATATCCCCAAAAGGGGAGTGACTTGGGATGGGGT
9 Rnd 1	GGCAGAAAAAGAATATCCCCAAAAGGGGAGTGACTTGGGATGGGGA
10 Rnd 1	GGCAGTAAAAGAATATCCCCAAAAGGGGAGTGACTTGGGATGGGGC
11 Rnd 1	GGCAGTAAAAGAATATCTCCAAAAGGAGAGTGACTTGGGATGAGGT
12 Rnd 1	GGCAGAAAAAGAATATCCCCAAAAGGGGAGTGACTTGGGATGGGGG
13 Rnd 1	GGCAGAAAAAGAATTTCTCCAAAAGGAGAGTGACTTGGGATGAGGT
14 Rnd 1	GGCAGTAAAAGAATTTCTCCAAAAGGAGAGTGACTTGGGATGAGGA
15 Rnd 1	GGCAGAAAAAGAATATCCCTAAAAAGGGAGTGACTTGGGATGGGTC
16 Rnd 1	GGCAGTAAAAGAATATCTCTAAAAAGAGAGTGACTTGGGATGAGTG
17 Rnd 1	GGCAGTAAAAGAATATCCTCAAAAGAGGAGTGACTTGGGATGGAGT
18 Rnd 1	GGAAGTAAAAGAATTTCCCTAAAAAGGGAGTGACTTGGGATGGGTT
19 Rnd 1	GGAAGTAAAAGAATTTCTCCAAAAGGAGAGTGACTTGGGATGAGGT
20 Rnd 1	GGCAGAAAAAGAATTTCCCCAAAAGGGGAGTGACTTGGGATGGGGG
21 Rnd 1	GGCAGAAAAAGAATATCCTTAAAAAAGGAGTGACTTGGGATGGTTT
22 Rnd 1	GGCAGTAAAAGAACATCTCCAAAAGGAGAGTGACTTGGGATGAGGA
23 Rnd 1	GGCAGTAAAAGAATATCTTCAAAAGGAGAGTGACTTGGGATGAGGA
24 Rnd 1	GGCAGTAAAAGCAAATCCTCAAAAGAGGAGTGACCTGGGATGGTGT
25 Rnd 1	GGCAGAAAAAGAATTTCCCTAAAAAGGGAGTGACCTGGGATGGGAG
1 Rnd 2	GGCAGAAAAAGAATTTCCCTAAAAAGGGAGTGACTTGGGATGGGTT
2 Rnd 2	GGCAGAAAAAGAATTTCCCCAAAAGGGGAGTGACTTGGGATGGGGT
3 Rnd 2	GGCAGTAAAAGAATTTCCCCAAAAGGGGAGTGACTTGGGATGGGGT
4 Rnd 2	GGCAGTAAAAGAATTTCTCCAAAAGGAGAGTGACTTGGGATGAGGT
5 Rnd 2	GGCAGAAAAAGAATATCCCCAAAAGGGGAGTGACTTGGGATGGGGT
6 Rnd 2	GGCAGAAAAAGAATATCTCCAAAAGGAGAGTGACTTGGGATGAGGT
7 Rnd 2	GGCAGAAAAAGAATATCCCCAAAAGGGGAGTGACTTGGGATGGGGA
8 Rnd 2	GGCAGAAAAAGAATATCTCCAAAAGGAGAGTGACTTGGGATGAGGA
9 Rnd 2	GGCAGAAAAAGAATATCCCTAAAAAGGGAGTGACTTGGGATGGGTT
10 Rnd 2	GGCAGAAAAAGAATATCTCCAAAAGGAGAGTGACTTGGGATGAGGC
11 Rnd 2	GGCAGAAAAAGAATATCCCTAAAAAGGGAGTGACTTGGGATGGGTA
12 Rnd 2	GGCAGAAAAAGAATATCCCTAAAAAGGGAGTGACTTGGGATGGGAT
13 Rnd 2	GGCAGAAAAAGAATTTCCCTAAAAAGGGAGTGACTTGGGATGGGTA
14 Rnd 2	GGCAGAAAAAGAATATCTCTAAAAAGAGAGTGACTTGGGATGAGTG
15 Rnd 2	GGCAGTAAAAGAATATCCCCAAAAGGGGAGTGACTTGGGATGGGGA
16 Rnd 2	GGCAGAAAAAGAATATCTCTAAAAAGAGAGTGACTTGGGATGAGTC
17 Rnd 2	GGCAGAAAAAGAATATCTCTAAAAAGAGAGTGACTTGGGATGAGAT
18 Rnd 2	GGCAGTAAAAGAATTTCTCCAAAAGGAGAGTGACTTGGGATGAGGA
19 Rnd 2	GGCAGTAAAAGAATATCTCTAAAAAGAGAGTGACTTGGGATGAGTT
20 Rnd 2	GGCAGTAAAAGAATATCTCTAAAAAGAGAGTGACTTGGGATGAGAT
21 Rnd 2	GGCAGAAAAAGAATATCTCCAAAAGGAGAGTGACTTGGGATGTGGT
22 Rnd 2	GGCAGAAAAAGAATATCCTTAAAAAAGGAGTGACTTGGGATGGTTG
23 Rnd 2	GGCAGAAAAAGAATATCTTCAAAAGGAGAGTGACTTGGGATGAGGT
24 Rnd 2	GGAAGTAAAAGAATTTCCCTAAAAAGGGAGTGACTTGGGATGGGTA
25 Rnd 2	GGAAGAAAAAGAATATCTCCAAAAGGAGAGTGACTTGGGATGTGGT
26 Rnd 2	GGCAGAAAAAGAATATCCTCAAAAGAGGAGTGACTTGGGATGGTGG
1 Rnd 3	GGCAGAAAAAGAATATCTCCAAAAGGAGAGTGACTTGGGATGAGGT
2 Rnd 3	GGCAGAAAAAGAATATCCCCAAAAGGGGAGTGACTTGGGATGGGGT
3 Rnd 3	GGCAGAAAAAGAATATCTCTAAAAAGAGAGTGACTTGGGATGAGTT
4 Rnd 3	GGCAGAAAAAGAATATCTCCAAAAGGAGAGTGACTTGGGATGAGGA
5 Rnd 3	GGCAGAAAAAGAATATCCCTAAAAAGGGAGTGACTTGGGATGGGTG
6 Rnd 3	GGCAGAAAAAGAATATCCCTAAAAAGGGAGTGACTTGGGATGGGTT
7 Rnd 3	GGCAGAAAAAGAATATCTCCAAAAGGAGAGTGACTTGGGATGAGGG
8 Rnd 3	GGCAGAAAAAGAATATCTCCAAAAGGAGAGTGACTTGGGATGAGGC
9 Rnd 3	GGCAGAAAAAGAATTTCCCTAAAAAGGGAGTGACTTGGGATGGGTT

10 Rnd 3	GGCAGTAAAAGAATATCTCCAAAAGGAGAGTGACTTGGGATGAGGT
11 Rnd 3	GGCAGAAAAAGAATATCTCTAAAAAGAGAGTGACTTGGGATGAGTA
12 Rnd 3	GGCAGAAAAAGAATATCCCCAAAAGGGGAGTGACTTGGGATGGGGA
13 Rnd 3	GGCAGAAAAAGAATATCCCTAAAAAGGGAGTGACTTGGGATGGGTC
14 Rnd 3	GGCAGAAAAAGAATTTCTCTAAAAAGAGAGTGACTTGGGATGAGTT
15 Rnd 3	GGCAGTAAAAGAATTTCCCTAAAAAGGGAGTGACTTGGGATGGGTT
16 Rnd 3	GGCAGTAAAAGAATATCTCCAAAAGGAGAGTGACTTGGGATGAGGA
17 Rnd 3	GGCAGTAAAAGAATTTCCCCAAAAGGGGAGTGACTTGGGATGGGGT
18 Rnd 3	GGCAGAAAAAGAATATCCCTAAAAAGGGAGTGACTTGGGATGGGAT
19 Rnd 3	GGCAGTAAAAGAATATCTCCAAAAGGAGAGTGACTTGGGATGAGGC
20 Rnd 3	GGCAGAAAAAGAATTTCCCTAAAAAGGGAGTGACTTGGGATGGGTG
21 Rnd 3	GGAAGAAAAAGAATATCCCCAAAAGGGGAGTGACTTGGGATGGGGT
22 Rnd 3	GGCAGTAAAAGAATATCCCTAAAAAGGGAGTGACTTGGGATGGGTC
23 Rnd 3	GGCAGAAAAAGAATTTCTCTAAAAAGAGAGTGACTTGGGATGAGTG
24 Rnd 3	GGCAGTAAAAGAATATCCCCAAAAGGGGAGTGACTTGGGATGGGGC
25 Rnd 3	GGAAGTAAAAGAATTTCCCTAAAAAGGGAGTGACTTGGGATGGGTT
26 Rnd 3	GGCAGAAAAAGAACATCTCTAAAAAGAGAGTGACTTGGGATGAGTC
1 Rnd 4	GGCAGAAAAAGAATATCTCCAAAAGGAGAGTGACTTGGGATGAGGT
2 Rnd 4	GGCAGAAAAAGAATATCCCCAAAAGGGGAGTGACTTGGGATGGGGT
3 Rnd 4	GGCAGAAAAAGAATATCTCTAAAAAGAGAGTGACTTGGGATGAGTT
4 Rnd 4	GGCAGAAAAAGAATATCCCTAAAAAGGGAGTGACTTGGGATGGGTG
5 Rnd 4	GGCAGAAAAAGAATATCCCTAAAAAGGGAGTGACTTGGGATGGGTT
6 Rnd 4	GGCAGAAAAAGAATATCTCTAAAAAGAGAGTGACTTGGGATGAGTG
7 Rnd 4	GGCAGAAAAAGAATATCTCCAAAAGGAGAGTGACTTGGGATGAGGA
8 Rnd 4	GGCAGAAAAAGAATATCTCCAAAAGGAGAGTGACTTGGGATGAGGG
9 Rnd 4	GGCAGTAAAAGAATATCTCCAAAAGGAGAGTGACTTGGGATGAGGT
10 Rnd 4	GGCAGAAAAAGAATATCTCCAAAAGGAGAGTGACTTGGGATGAGGC
11 Rnd 4	GGCAGAAAAAGAATATCTCTAAAAAGAGAGTGACTTGGGATGAGTC
12 Rnd 4	GGCAGAAAAAGAATATCCCCAAAAGGGGAGTGACTTGGGATGGGGG
13 Rnd 4	GGCAGAAAAAGAATATCTCTAAAAAGAGAGTGACTTGGGATGAGTA
14 Rnd 4	GGCAGAAAAAGAATTTCCCCAAAAGGGGAGTGACTTGGGATGGGGT
15 Rnd 4	GGCAGAAAAAGAATATCTCTAAAAAGAGAGTGACTTGGGATGAGAT
16 Rnd 4	GGCAGAAAAAGAATTTCTCCAAAAGGAGAGTGACTTGGGATGAGGT
17 Rnd 4	GGCAGAAAAAGAATATCCCCAAAAGGGGAGTGACTTGGGATGGGGC
18 Rnd 4	GGCAGTAAAAGAATATCCCTAAAAAGGGAGTGACTTGGGATGGGTG
19 Rnd 4	GGCAGTAAAAGAATTTCTCTAAAAAGAGAGTGACTTGGGATGAGTT
20 Rnd 4	GGAAGAAAAAGAATATCTCCAAAAGGAGAGTGACTTGGGATGAGGT
21 Rnd 4	GGCAGTAAAAGAATATCTCTAAAAAGAGAGTGACTTGGGATGAGTA
22 Rnd 4	GGCAGTAAAAGAATTTCCCTAAAAAGGGAGTGACTTGGGATGGGTG
23 Rnd 4	GGCAGAAAAAGAATATCCCTAAAAGGGGAGTGACTTGGGATGGGGT
24 Rnd 4	GGAAGAAAAAGAATATCTCCAAAAGGAGAGTGACTTGGGATGAGGC
25 Rnd 4	GGCAGAAAAAGAATATCTCTAAAAGGAGAGTGACTTGGGATGAGGC
**Secondary structure selection top hits (cpm)**	
1 Rnd 1	GGCAGAAAAAGAATTTCCCTAAAAAGGGAGTGACTTGGGATGGGTG
2 Rnd 1	GGCAGTAAAAGAATTTCTCTAAAAAGAGAGTGACTTGGGATGAGTT
3 Rnd 1	GGCAGTAAAAGAATTTCCCTAAAAAGGGAGTGACTTGGGATGGGTG
4 Rnd 1	GGCAGTAAAAGAATTTCCCTAAAAAGGGAGTGACTTGGGATGGGTT
5 Rnd 1	GGCAGAAAAAGAATTTCCCTAAAAAGGGAGTGACTTGGGATGGGTT
6 Rnd 1	GGCAGTAAAAGAATTTCTCTAAAAAGAGAGTGACTTGGGATGAGTG
7 Rnd 1	GGCAGAAAAAGAATTTCTCTAAAAAGAGAGTGACTTGGGATGAGTT
8 Rnd 1	GGCAGAAAAAGAATATCTCTAAAAGGAGAGTGACTTGGGATGAGGT
9 Rnd 1	GGCAGAAAAAGAATATCTCTAAAAAGAGAGTGACTTGGGATGAGTG
10 Rnd 1	GGCAGAAAAAGAATATTCCTAAAAAGGGAGTGACTTGGGATGGGTG
1 Rnd 2	GGCAGAAAAAGAATTTCCCTAAAAAGGGAGTGACTTGGGATGGGTT
2 Rnd 2	GGCAGAAAAAGAATATCTCTAAAAAGAGAGTGACTTGGGATGAGTG
3 Rnd 2	GGCAGAAAAAGAATTTCCCTAAAAAGGGAGTGACTTGGGATGGGTG

4 Rnd 2	GGCAGAAAAAGAATTTCTCTAAAAAGAGAGTGACTTGGGATGAGTT
5 Rnd 2	GGCAGTAAAAGAATTTCCCTAAAAAGGGAGTGACTTGGGATGGGTT
6 Rnd 2	GGCAGAAAAAGAATATCTCTAAAAAGAGAGTGACTTGGGATGAGTT
7 Rnd 2	GGCAGAAAAAGAATATCCCTAAAAAGGGAGTGACTTGGGATGGGTG
8 Rnd 2	GGCAGAAAAAGAATTTCTCCAAAAGGAGAGTGACTTGGGATGAGGT
9 Rnd 2	GGCAGTAAAAGAATTTCTCTAAAAAGAGAGTGACTTGGGATGAGTT
10 Rnd 2	GGCAGAAAAAGAATATCTCCAAAAGGAGAGTGACTTGGGATGAGGG
1 Rnd 3	GGCAGAAAAAGAATATCTCTAAAAAGAGAGTGACTTGGGATGAGTG
2 Rnd 3	GGCAGAAAAAGAATATCTCTAAAAAGAGAGTGACTTGGGATGAGTT
3 Rnd 3	GGCAGAAAAAGAATATCTCCAAAAGGAGAGTGACTTGGGATGAGGT
4 Rnd 3	GGCAGAAAAAGAATATCCCTAAAAAGGGAGTGACTTGGGATGGGTG
5 Rnd 3	GGCAGAAAAAGAATTTCTCTAAAAAGAGAGTGACTTGGGATGAGTT
6 Rnd 3	GGCAGAAAAAGAATATCTCCAAAAGGAGAGTGACTTGGGATGAGGG
7 Rnd 3	GGCAGAAAAAGAATATCTCTAAAAAGAGAGTGACTTGGGATGAGTA
8 Rnd 3	GGCAGAAAAAGAATTTCTCCAAAAGGAGAGTGACTTGGGATGAGGT
9 Rnd 3	GGCAGAAAAAGAATATCTCTAAAAAGAGAGTGACTTGGGATGAGTC
10 Rnd 3	GGCAGAAAAAGAATATCCCTAAAAAGGGAGTGACTTGGGATGGGTT
1 Rnd 4	GGCAGAAAAAGAATATCTCTAAAAAGAGAGTGACTTGGGATGAGTG
2 Rnd 4	GGCAGAAAAAGAATATCTCTAAAAAGAGAGTGACTTGGGATGAGTT
3 Rnd 4	GGCAGAAAAAGAATATCTCCAAAAGGAGAGTGACTTGGGATGAGGT
4 Rnd 4	GGCAGAAAAAGAATATCCCTAAAAAGGGAGTGACTTGGGATGGGTG
5 Rnd 4	GGCAGAAAAAGAATATCTCCAAAAGGAGAGTGACTTGGGATGAGGG
6 Rnd 4	GGCAGAAAAAGAATATCCCTAAAAAGGGAGTGACTTGGGATGGGTT
7 Rnd 4	GGCAGAAAAAGAATATCTCTAAAAAGAGAGTGACTTGGGATGAGTC
8 Rnd 4	GGCAGAAAAAGAATATCTCTAAAAGGAGAGTGACTTGGGATGAGGT
9 Rnd 4	GGCAGAAAAAGAATATCTCTAAAAAGAGAGTGACTTGGGATGAGTA
10 Rnd 4	GGCAGAAAAAGAATATCCCCAAAAGGGGAGTGACTTGGGATGGGGT
**Secondary structure selection top hits (enrichment)**	
1 Rnd 1	GGCAGTAAAAGAATTTCCCTAAAAAGGGAGTGACTTGGGATGGGTT
2 Rnd 1	GGCAGTAAAAGAATTTCCCCAAAAGGGGAGTGACTTGGGATGGGGT
3 Rnd 1	GGCAGTAAAAGAATTTCCCTAAAAAGGGAGTGACTTGGGATGGGTG
4 Rnd 1	GGCAGTAAAAGAATTTCCCTAAAAAGGGAGTGACTTGGGATGGGTA
5 Rnd 1	GGCAGTAAAAGAATTTCTCCAAAAGGAGAGTGACTTGGGATGAGGT
6 Rnd 1	GGCAGAAAAAGAATTTCCCTAAAAAGGGAGTGACTTGGGATGGGTT
7 Rnd 1	GGCAGAAAAAGAATTTCCCCAAAAGGGGAGTGACTTGGGATGGGGT
8 Rnd 1	GGCAGAAAAAGAATTTCCCTAAAAAGGGAGTGACTTGGGATGGGTG
9 Rnd 1	GGCAGAAAAAGAATATCCCCAAAAGGGGAGTGACTTGGGATGGGG
10 Rnd 1	GGCAGTAAAAGAATATCCCCAAAAGGGGAGTGACTTGGGATGGGGT
1 Rnd 2	GGCAGAAAAAGAATTTCCCTAAAAAGGGAGTGACTTGGGATGGGTT
2 Rnd 2	GGCAGTAAAAGAATTTCCCTAAAAAGGGAGTGACTTGGGATGGGTT
3 Rnd 2	GGCAGAAAAAGAATTTCCCCAAAAGGGGAGTGACTTGGGATGGGGT
4 Rnd 2	GGCAGTAAAAGAATTTCCCCAAAAGGGGAGTGACTTGGGATGGGGT
5 Rnd 2	GGCAGTAAAAGAATTTCTCCAAAAGGAGAGTGACTTGGGATGAGGT
6 Rnd 2	GGCAGAAAAAGAATATCCCCAAAAGGGGAGTGACTTGGGATGGGGT
7 Rnd 2	GGCAGAAAAAGAATATCTCCAAAAGGAGAGTGACTTGGGATGAGGT
8 Rnd 2	GGCAGAAAAAGAATATCCCTAAAAAGGGAGTGACTTGGGATGGGTG
9 Rnd 2	GGCAGAAAAAGAATTTCTCCAAAAGGAGAGTGACTTGGGATGAGGT
10 Rnd 2	GGCAGAAAAAGAATATCCCCAAAAGGGGAGTGACTTGGGATGGGGA
1 Rnd 3	GGCAGAAAAAGAATATCTCCAAAAGGAGAGTGACTTGGGATGAGGT
2 Rnd 3	GGCAGAAAAAGAATATCCCCAAAAGGGGAGTGACTTGGGATGGGGT
3 Rnd 3	GGCAGAAAAAGAATATCTCTAAAAAGAGAGTGACTTGGGATGAGTT
4 Rnd 3	GGCAGAAAAAGAATATCTCCAAAAGGAGAGTGACTTGGGATGAGGA
5 Rnd 3	GGCAGAAAAAGAATATCCCTAAAAAGGGAGTGACTTGGGATGGGTG
6 Rnd 3	GGCAGAAAAAGAATATCTCTAAAAAGAGAGTGACTTGGGATGAGTG
7 Rnd 3	GGCAGAAAAAGAATATCCCTAAAAAGGGAGTGACTTGGGATGGGTT
8 Rnd 3	GGCAGAAAAAGAATATCTCCAAAAGGAGAGTGACTTGGGATGAGGG

9 Rnd 3	GGCAGAAAAAGAATATCTCCAAAAGGAGAGTGACTTGGGATGAGGC
10 Rnd 3	GGCAGAAAAAGAATTTCTCCAAAAGGAGAGTGACTTGGGATGAGGT
1 Rnd 4	GGCAGAAAAAGAATATCTCCAAAAGGAGAGTGACTTGGGATGAGGT
2 Rnd 4	GGCAGAAAAAGAATATCCCCAAAAGGGGAGTGACTTGGGATGGGGT
3 Rnd 4	GGCAGAAAAAGAATATCTCTAAAAAGAGAGTGACTTGGGATGAGTT
4 Rnd 4	GGCAGAAAAAGAATATCCCTAAAAAGGGAGTGACTTGGGATGGGTG
5 Rnd 4	GGCAGAAAAAGAATATCCCTAAAAAGGGAGTGACTTGGGATGGGTT
6 Rnd 4	GGCAGAAAAAGAATATCTCTAAAAAGAGAGTGACTTGGGATGAGTG
7 Rnd 4	GGCAGAAAAAGAATATCTCCAAAAGGAGAGTGACTTGGGATGAGGA
8 Rnd 4	GGCAGAAAAAGAATATCTCCAAAAGGAGAGTGACTTGGGATGAGGG
9 Rnd 4	GGCAGTAAAAGAATATCTCCAAAAGGAGAGTGACTTGGGATGAGGT
10 Rnd 4	GGCAGAAAAAGAATATCCCTAAAAAGGGAGTGACTTGGGATGGGTC
Hit 1	GGCAGAAAAAGAATATCTCCAAAAGGAGAGTGACTTGGGATGAGGT
Negative control selection library	GDARNRAAAAGMWDWKCCCCAAAARRRGRGTGACYTSRNATRRGNGA ACGCTTGCTCCGGTCTGATGT
Negative control selection splint	WHWKCTTTTYNYTHCCGCAGGGACTTCAGTGGATC
**Negative control selection top hits (cpm)**	
1	GGAATAAAAAGATTTGCCCCAAAAAAAGAGTGACTTCAGATGAGTG
2	GGAAGAAAAAGAATTTCCCCAAAAGGGGAGTGACTTGGGATGGGGG
3	GGAAGAAAAAGAAAAGCCCCAAAAAAAGGGGTGACTTCATATAGGTG
4	GGAAAGAAAAGATTTTCCCCAAAAAAAGAGTGACTTGAAATGAGTG
5	GGAAAGAAAAGATTATCCCCAAAAAAAGAGTGACTTCGCATAAGTG
6	GGAAAAAAAAGCTTTGCCCCAAAAAAAGAGTGACTTCATATAAGCG
7	GGAAAAAAAAGATTTTCCCCAAAAAGAGGGTGACTTGATATAGGTG
8	GGAAAAAAAAGATTTTCCCCAAAAAAAGAGTGACTTCGTATGGGAG
9	GGAAAAAAAAGATTTTCCCCAAAAAAAGAGTGACTTCATATAGGTG
10	GGAAAAAAAAGATTTTCCCCAAAAAAAGAGTGACTTCATATAAGTG
Spacer selection library	GGCAGANNNNGAATATCTCCNNNNGGAGAGTGACTTGGGATGAGGTAA GCCTGGAAGTAACTCTGGTT
Fwd2	TACGAGAGATGAGGTAAGCC
Rev2	AACCAGAGTTACTTCCAGGC
Splint2	TATTCNNNNTCTGCCGGCTTACCTCATCTCTCGTA
**Spacer selection fitness landscape analysis (cpm)**	
1	GGCAGATCTTGAATATCTCCTTTTGGAGAGTGACTTGGGATGAGGT
2	GGCAGATTTTGAATATCTCCTTTTGGAGAGTGACTTGGGATGAGGT
3	GGCAGATCTTGAATATCTCCTCTTGGAGAGTGACTTGGGATGAGGT
4	GGCAGATCTTGAATATCTCCTATTGGAGAGTGACTTGGGATGAGGT
5	GGCAGATCTTGAATATCTCCTTTGGGAGAGTGACTTGGGATGAGGT
6	GGCAGATTTTGAATATCTCCATTTGGAGAGTGACTTGGGATGAGGT
7	GGCAGATCTTGAATATCTCCTATAGGAGAGTGACTTGGGATGAGGT
8	GGCAGATTTTGAATATCTCCTTTGGGAGAGTGACTTGGGATGAGGT
9	GGCAGATCTTGAATATCTCCATTAGGAGAGTGACTTGGGATGAGGT
10	GGCAGATTTTGAATATCTCCTTAAGGAGAGTGACTTGGGATGAGGT
11	GGCAGATCTTGAATATCTCCTGTGGAGAGTGACTTGGGATGAGGT
12	GGCAGATTTTGAATATCTCCTTGAGGAGAGTGACTTGGGATGAGGT
13	GGCAGATTTTGAATATCTCCATGTGGAGAGTGACTTGGGATGAGGT
14	GGCAGATCTTGAATATCTCCAGATGGAGAGTGACTTGGGATGAGGT
15	GGCAGATCTTGAATATCTCCTCAGGGAGAGTGACTTGGGATGAGGT

16	GGCAGACTTAGAATATCTCCAGTAGGAGAGTGACTTGGGATGAGGT
17	GGCAGATAATGAATATCTCCTTTTGGAGAGTGACTTGGGATGAGGT
18	GGCAGATCCTGAATATCTCCAATGGGAGAGTGACTTGGGATGAGGT
19	GGCAGACTTTGAATATCTCCTCAGGGAGAGTGACTTGGGATGAGGT
20	GGCAGATCCAGAATATCTCCTAAAGGAGAGTGACTTGGGATGAGGT
21	GGCAGATCCAGAATATCTCCTAGGGGAGAGTGACTTGGGATGAGGT
22	GGCAGATGTTGAATATCTCCGCGTGGAGAGTGACTTGGGATGAGGT
23	GGCAGACAACGAATATCTCCTCACGGAGAGTGACTTGGGATGAGGT
**Spacer selection fitness landscape analysis (enrichment)**	
1	GGCAGACACAGAATATCTCCCTCGGGAGAGTGACTTGGGATGAGGT
2	GGCAGACACAGAATATCTCCCAGGGGAGAGTGACTTGGGATGAGGT
3	GGCAGACACAGAATATCTCCCACGGGAGAGTGACTTGGGATGAGGT
4	GGCAGACACAGAATATCTCCCCGAGGAGAGTGACTTGGGATGAGGT
5	GGCAGACACAGAATATCTCCTCGAGGAGAGTGACTTGGGATGAGGT
6	GGCAGACACAGAATATCTCCACATGGAGAGTGACTTGGGATGAGGT
7	GGCAGACACAGAATATCTCCTCCAGGAGAGTGACTTGGGATGAGGT
8	GGCAGACACAGAATATCTCCATATGGAGAGTGACTTGGGATGAGGT
9	GGCAGACACAGAATATCTCCGATTGGAGAGTGACTTGGGATGAGGT
10	GGCAGATCCTGAATATCTCCCGGGGGAGAGTGACTTGGGATGAGGT
11	GGCAGACACAGAATATCTCCATGAGGAGAGTGACTTGGGATGAGGT
12	GGCAGACTCAGAATATCTCCTGTTGGAGAGTGACTTGGGATGAGGT
13	GGCAGATCTTGAATATCTCCTCATGGAGAGTGACTTGGGATGAGGT
14	GGCAGATCCTGAATATCTCCCCGGGGAGAGTGACTTGGGATGAGGT
15	GGCAGACACAGAATATCTCCTTGCGGAGAGTGACTTGGGATGAGGT
16	GGCAGACATAGAATATCTCCTGAAGGAGAGTGACTTGGGATGAGGT
17	GGCAGACTTAGAATATCTCCTCGTGGAGAGTGACTTGGGATGAGGT
18	GGCAGACTTAGAATATCTCCTAACGGAGAGTGACTTGGGATGAGGT
19	GGCAGATCCAGAATATCTCCGTCGGGAGAGTGACTTGGGATGAGGT
20	GGCAGATCTCGAATATCTCCATCAGGAGAGTGACTTGGGATGAGGT
21	GGCAGATCGTGAATATCTCCTTCGGGAGAGTGACTTGGGATGAGGT
22	GGCAGACCCTGAATATCTCCCCGAGGAGAGTGACTTGGGATGAGGT
23	GGCAGACGTTGAATATCTCCAGAGGGAGAGTGACTTGGGATGAGGT
24	GGCAGACGCCGAATATCTCCGTGGGGAGAGTGACTTGGGATGAGGT
25	GGCAGAATAAGAATATCTCCTCGAGGAGAGTGACTTGGGATGAGGT
**Spacer selection top hits (enrichment)**	
1	GGCAGACACAGAATATCTCCCTCGGGAGAGTGACTTGGGATGAGGT
2	GGCAGACACAGAATATCTCCTTCAGGAGAGTGACTTGGGATGAGGT
3	GGCAGACACAGAATATCTCCTACAGGAGAGTGACTTGGGATGAGGT
4	GGCAGACACAGAATATCTCCCAGGGGAGAGTGACTTGGGATGAGGT
5	GGCAGACACAGAATATCTCCCCCGGGAGAGTGACTTGGGATGAGGT
6	GGCAGACACAGAATATCTCCCTTGGGAGAGTGACTTGGGATGAGGT
7	GGCAGACACAGAATATCTCCCCGGGGAGAGTGACTTGGGATGAGGT
8	GGCAGACACAGAATATCTCCCACGGGAGAGTGACTTGGGATGAGGT
9	GGCAGACACAGAATATCTCCTGCAGGAGAGTGACTTGGGATGAGGT
10	GGCAGACACAGAATATCTCCCGGGGGAGAGTGACTTGGGATGAGGT
**Core and Spacer combination variants**	
1	GGCAGACACAGAATATCTCCCGGGGGAGAGTGACTTGGGATGAGGT
2	GGCAGACACAGAATATCTCCTACAGGAGAGTGACTTGGGATGAGGT
3	GGCAGACACAGAATATCTCCTTCAGGAGAGTGACTTGGGATGAGGT
4	GGCAGACACAGAATATCTCCCTCGGGAGAGTGACTTGGGATGAGGT
5	GGCAGACACAGAATATCTCCCAGGGGAGAGTGACTTGGGATGAGGT
6	GGCAGACACAGAATTTCCCTCGGGAGGGAGTGACTTGGGATGGGTT
7	GGCAGACACAGAATTTCCCTTACAAGGGAGTGACTTGGGATGGGTT
8	GGCAGACACAGAATTTCCCTTTCAAGGGAGTGACTTGGGATGGGTT

9	GGCAGACACAGAATTTCCCTCTCGAGGGAGTGACTTGGGATGGGTT
10	GGCAGACACAGAATTTCCCTCAGGAGGGAGTGACTTGGGATGGGTT
11	GGCAGACACAGAATATCTCTCGGGAGAGAGTGACTTGGGATGAGTA
12; also Hypernova	GGCAGACACAGAATATCTCTTACAAGAGAGTGACTTGGGATGAGTA
13	GGCAGACACAGAATATCTCTTTCAAGAGAGTGACTTGGGATGAGTA
14	GGCAGACACAGAATATCTCTCTCGAGAGAGTGACTTGGGATGAGTA
15	GGCAGACACAGAATATCTCTCAGGAGAGAGTGACTTGGGATGAGTA
16	GGCAGACACAGAATATCTCCCGGGGGAGAGTGACTTGGGATGAGGG
17	GGCAGACACAGAATATCTCCTACAGGAGAGTGACTTGGGATGAGGG
18	GGCAGACACAGAATATCTCCTTCAGGAGAGTGACTTGGGATGAGGG
19	GGCAGACACAGAATATCTCCCTCGGGAGAGTGACTTGGGATGAGGG
20	GGCAGACACAGAATATCTCCCAGGGGAGAGTGACTTGGGATGAGGG
21	GGCAGACACAGAATATCTCTCGGGAGAGAGTGACTTGGGATGAGTG
22	GGCAGACACAGAATATCTCTTACAAGAGAGTGACTTGGGATGAGTG
23	GGCAGACACAGAATATCTCTTTCAAGAGAGTGACTTGGGATGAGTG
24	GGCAGACACAGAATATCTCTCTCGAGAGAGTGACTTGGGATGAGTG
25	GGCAGACACAGAATATCTCTCAGGAGAGAGTGACTTGGGATGAGTG
Splint for HN	TATTCTGTGTCTGCCTGATACTACACCTGAGCGGT
rHN	AAAArCGGCAGACACAGAATATCTCTTACAAGAGAGTGACTTGGGATGAGTA
rSN	AAAArCGGAAGAAAAAGAATATCCCCAAAAGGGGAGTGACTTGGGATGGGGG
aSN	GGAAGAAAAAGAATATCTCCAAAAGGAGAGTGACTTGGGATGAGGG
